# Pathway Crosstalk and Metabolic Relay in the Phenylpropanoid–Carotenoid Axis Orchestrate Petal Coloration in *Kalanchoe blossfeldiana*

**DOI:** 10.3390/genes17080934

**Published:** 2026-08-11

**Authors:** Mei Zhang, Siyang Duan, Ji Zhang, Riwen Fei, Xiuting Zhao, Changbo Ji, Li Liu

**Affiliations:** 1 College of Science, Liaodong University, Dandong 118003, China; zhangmei@liaodongu.edu.cn; 2 College of Agriculture, Liaodong University, Dandong 118003, China; 923014@liaodongu.edu.cn (R.F.); 924080@liaodongu.edu.cn (X.Z.); jizhangbo@liaodongu.edu.cn (C.J.); 3 Taizhixing Park Management Co., Ltd., Tianjin 300457, China; saberzj940501@gmail.com

**Keywords:** *Kalanchoe blossfeldiana*, flower color, transcriptome, metabolome

## Abstract

**Background:** Flower color is a primary determinant of ornamental value in flowering plants. Although the biosynthetic pathways of major petal pigments have been extensively documented, the systems-level mechanisms coordinating color diversification across multiple cultivars of *Kalanchoe blossfeldiana* remain less understood. This study aimed to establish a comparative metabolomic and transcriptomic framework to elucidate these mechanisms. **Methods:** Petal tissues were collected from five cultivars with white, yellow, orange, red, and purple–red flowers at the full-bloom stage. Metabolomics was performed for flavonoids and carotenoids using UPLC-MS/MS, and transcriptome sequencing was conducted via Illumina NovaSeq 6000. Integrative analysis of metabolome and transcriptome data, together with weighted gene co-expression network analysis, was employed to identify key metabolites, differentially expressed genes, and associated regulatory networks. Quantitative PCR was used to validate the expression patterns of key structural genes. **Results:** Petal color variation was shaped by coordinated flux partitioning between flavonoid and carotenoid networks rather than by isolated activation of single pathways. A metabolic relay pattern was observed in the phenylpropanoid–flavonoid axis, wherein shared upstream activation fed divergent downstream branches that directed accumulation toward distinct anthocyanin or flavonol profiles. Notably, yellow pigmentation relied more heavily on flavonoid flux than on carotenoid accumulation, and orange coloration arose from the synergistic co-accumulation of multiple pigment classes. Transcriptional module analysis identified gene networks associated with specific color phenotypes. **Conclusions:** These findings provide a systems-level understanding of pathway crosstalk in flower color formation and offer candidate targets for molecular breeding in *K. blossfeldiana* and related ornamental plants.

## 1. Introduction

Flower color represents one of the most crucial ornamental traits in plants. It not only determines the interactions between plants and pollinators but also serves as a key quality indicator for competitiveness in the ornamental plant market [1,2]. In nature, the flower colors of angiosperms cover a wide spectrum, ranging from yellow, orange, and red to purple and blue. This diversity mainly results from the accumulation and interaction of different types of pigments in petals [1,3]. For the ornamental horticulture industry, flower color continues to be the primary target trait in cultivar breeding [4]. *K. blossfeldiana*, which belongs to the Crassulaceae family and the Kalanchoe genus, is native to Madagascar. It has become one of the world’s most significant potted flowering plants because of its long blooming period, compact growth habit, and low maintenance requirements. According to the statistics from Royal FloraHolland, Kalanchoe ranked fourth in global sales volume in 2020, with annual sales surpassing 61 million euros [5]. After nearly a century of hybrid breeding, numerous cultivated cultivars with diverse flower colors, such as red, pink, yellow, orange, and white, have been developed. Nevertheless, the molecular mechanisms underlying these color variations remain unclear, which substantially hinders marker-assisted breeding.

The color of plant petals is predominantly determined by the composition and relative abundance of 3 categories of pigments, which are anthocyanins, flavonoids, and carotenoids [6,7]. Anthocyanins are water-soluble flavonoid compounds featuring a flavan backbone [8,9]. Through modifications such as glycosylation and acylation, they form stable glycosides that serve as the primary pigments responsible for red, purple, and blue flower colors [10,11]. Based on the degree of hydroxylation in the B ring, anthocyanins can be categorized into six major aglycones: pelargonidin (Pg), cyanidin (Cy), delphinidin (Dp), peonidin (Pn), petunidin (Pt), and malvidin (Mv) [12]. Flavonoids encompass flavones, flavonols, and isoflavones, which are typically colorless or pale yellow [13,14,15]. They not only modify flower color saturation and hue through copigmentation but also directly affect petal coloration via their own accumulation [16,17,18]. Carotenoids are lipid-soluble terpenoid pigments spanning from yellow to red, frequently coexisting with anthocyanins in petals or independently dictating yellow/orange hues [19]. Previous research has indicated that *K. blossfeldiana* petals contain all three types of pigments—anthocyanins, flavonoids, and carotenoids [20]. Nevertheless, the accumulation patterns, relative contributions, and metabolic differentiation of these pigments across different flower color cultivars remain inadequately understood.

The biosynthesis of anthocyanins and flavonoids begins with the phenylpropanoid pathway [21]. In this pathway, phenylalanine is transformed into cinnamic acid by phenylalanine ammonia-lyase (PAL), and then it is converted to 4-coumaroyl-CoA through the actions of cinnamate 4-hydroxylase (C4H) and 4-coumarate:CoA ligase (4CL). Chalcone synthase (CHS) catalyzes the synthesis of chalcone from this precursor. Subsequently, chalcone isomerase (CHI) isomerizes chalcone into flavanone, which then enters the core flavonoid pathway [22,23]. Flavanone 3-hydroxylase (F3H) catalyzes the generation of dihydrokaempferol, which serves as a crucial branching point. On one branch, dihydroflavonol 4-reductase (DFR) and anthocyanidin synthase (ANS) convert dihydrokaempferol into colorless anthocyanidins, which is then glycosylated by UDP-glucose:flavonoid 3-O-glucosyltransferase (UFGT) to form stable anthocyanins. On the other hand, flavonol synthase (FLS) directs dihydrokaempferol toward the flavonol synthesis pathway. The flux distribution at the F3H branching point is regarded as a critical factor determining the relative accumulation of anthocyanins and flavonols [24,25]. In the carotenoid pathway, isopentenyl diphosphate (IPP) and dimethylallyl diphosphate (DMAPP) are condensed by phytoene synthase (PSY) to form phytoene [26]. Phytoene then undergoes successive dehydrogenation processes by phytoene desaturase (PDS) and ζ-carotene desaturase (ZDS) to generate lycopene. Lycopene is then cyclized by lycopene cyclase (LCY) and further modified by β-carotene hydroxylase and other enzymes to yield end products such as xanthophylls and β-carotene [27,28,29,30]. The final accumulation and coloration of these three pigment classes in petals are collectively determined by the expression levels of these structural genes, their enzymatic activities, and the regulation by transcription factors, such as MYB, bHLH, and WD40, which form the MYB-bHLH-WD40 (MBW) complex [31,32,33]. Therefore, comparing the expression differences of these key genes among different flower color cultivars and their correlation with metabolite accumulation is an effective approach to uncovering the molecular basis of flower color diversification [34].

In recent years, the integrative analysis of metabolomics and transcriptomics has emerged as a mainstream approach for elucidating the mechanisms underlying flower color formation in plants [35,36,37]. In various ornamental plant species, including chrysanthemums, roses, peonies, and irises, researchers have effectively identified key pigment metabolites, structural genes, and transcription factors associated with flower color variation by integrating metabolomic and transcriptomic data [38,39,40]. In *K. blossfeldiana*, Feng et al. were the first to employ integrated metabolomics and transcriptomics to uncover pigment differences between the red and yellow regions within the petals of the same bicolor cultivar [20]. They found that the anthocyanin accumulation in the red region was approximately 19 times higher than that in the yellow region and identified potential positive regulators of anthocyanin biosynthesis, such as KbMYB2 and KbbHLH1. Nevertheless, this study was solely focused on intravarietal spatial color differences and utilized samples with a uniform genetic background, and thus could not explain the systematic differentiation mechanisms among different flower color cultivars. Furthermore, there is still a dearth of multi-omics studies comparing multiple independent flower color cultivars of *K. blossfeldiana*, especially regarding comprehensive evaluations of how the anthocyanin, flavonoid, and carotenoid pathways contribute either synergistically or independently across different cultivars. Therefore, establishing a comparative framework that encompasses multiple typical flower colors and simultaneously analyzes the divergence patterns of the anthocyanin, flavonoid, and carotenoid pathways is of significant theoretical and practical importance for understanding the molecular basis of flower color diversity in *K. blossfeldiana*.

This study utilized 5 cultivars of *K. blossfeldiana* with distinct flower colors. Petal tissues were collected at the peak blooming stage, and widely targeted metabolomics analysis based on scheduled multiple reaction monitoring (MRM) was carried out for flavonoids and carotenoids. Concurrently, transcriptome sequencing and targeted qPCR validation were conducted, thereby establishing a multi-layer integrative analytical framework of “metabolites–transcripts–gene expression”. Specifically, by comparing the differentially accumulated metabolites (DAMs) and differentially expressed genes (DEGs) among the five cultivars, the key pigment metabolites driving color variation and their pathway enrichment characteristics were identified. Transcriptional analysis was applied to screen the critical structural genes and regulatory factors in the three pigment biosynthetic pathways, clarifying their expression divergence patterns across different flower color cultivars. Moreover, through gene–metabolite correlation analysis, co-enrichment pathway mapping, and association network construction, the core regulatory node genes underlying color variation were discovered. Finally, qPCR was employed to independently validate six key color-related genes (PAL, CHS, CHI, FLS, UFGT, and ZDS) to ensure data reliability. This study aims to systematically elucidate the metabolic differentiation mechanisms underlying flower color variation in *K. blossfeldiana* from three levels: metabolite profiles, gene expression, and regulatory networks. It also aims to clarify the relative contributions and synergistic relationships among the anthocyanin, flavonoid, and carotenoid pathways in flower color formation and to provide theoretical foundations and candidate targets for the molecular breeding of flower color in *K. blossfeldiana* and other ornamental plants.

## 2. Materials and Methods

### 2.1. Plant Materials

**Figure 1:**
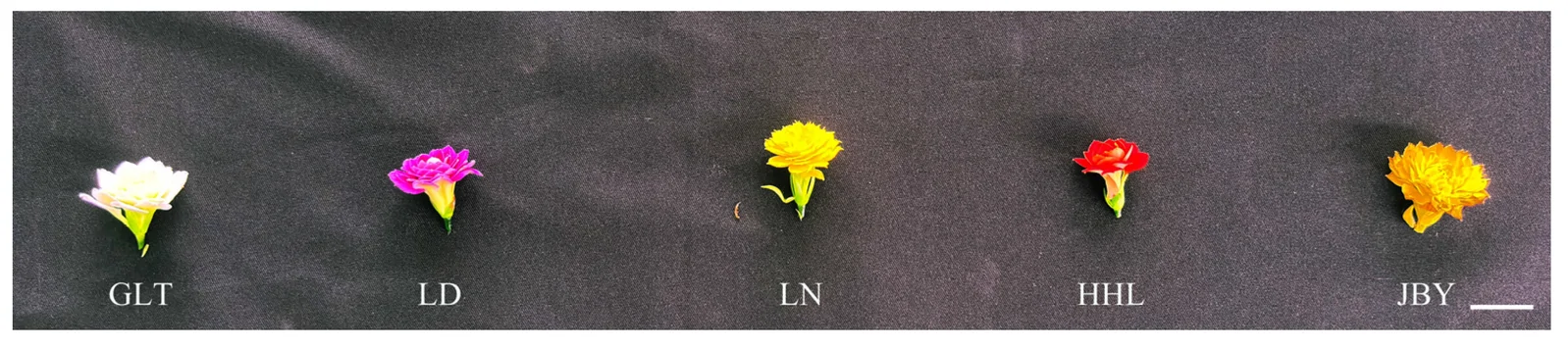
Five cultivars of *K. blossfeldiana* with different flower colors. The bar is 1.0 cm.

Five distinct cultivars of *K. blossfeldiana* with different flower colors were chosen and labeled as GLT (white), LD (purple–red), LN (yellow), HHL (red) and JBY (orange) (Figure 1). At the fully blooming stage, three healthy plants of each cultivated variety were randomly selected. Each plant was regarded as an independent biological replicate sample. Thus, each cultivated variety produced three groups of biological replicates, totaling 15 samples. For each group of biological replicates, 1 g of fully expanded petals was randomly collected from the same plant and combined into one sample. The petals were promptly flash-frozen in liquid nitrogen and stored at −80 °C for subsequent metabolomics, transcriptomics, and qPCR analyses.

### 2.2. Metabolite Extraction and UPLC-MS/MS Analysis

A widely targeted metabolomics strategy was employed in this study: all samples were analyzed directly using scheduled MRM-based acquisition, and no untargeted screening step was performed prior to targeted MRM analysis. Metabolite analysis encompassed two categories: flavonoids (comprising anthocyanins, flavones, flavonols, and their derivatives) and carotenoids. All samples were subjected to freeze-drying, followed by grinding into powder (at 30 Hz for 1.5 min), and subsequently stored at −80 °C for subsequent utilization. For the extraction of flavonoids, 20 mg of freeze-dried powder was mixed with 0.5 mL of a 70% methanol solution (Macklin, Shanghai, China) containing an internal standard (L-2-chlorophenylalanine, 0.4 mg/L), and then subjected to sonication for 30 min. The mixture was centrifuged at 12,000× *g* for 5 min at 4 °C; the supernatant was filtered through a 0.22 μm membrane filter and transferred to sample vials for analysis. For the extraction of carotenoids, 50 mg of freeze-dried powder was combined with 0.5 mL of a mixed solvent (n-hexane(Xilong, Shenzhen, China): acetone(Xilong, Shenzhen, China): ethanol (Xilong, Shenzhen, China) = 1:1:1, *v*/*v*/*v*, containing 0.01% BHT (Macklin, Shanghai, China)), vortexed at room temperature for 20 min, and centrifuged at 12,000 rpm for 5 min at 4 °C. The supernatant was collected, and the residue was extracted a second time. The two supernatants were combined, dried under vacuum, reconstituted in 150 μL of dichloromethane (Xilong, Shenzhen, China), filtered through a 0.22 μm membrane filter, and prepared for analysis.

Flavonoids and carotenoids were analyzed on the same UPLC-MS/MS platform, but with different columns and ion sources. Regarding flavonoids, the UPLC conditions were as follows: the column was Waters ACQUITY UPLC HSS T3 C18 (1.8 μm, 100 mm × 2.1 mm) (Waters, Milford, MA, USA); mobile phase A consisted of water containing 0.05% formic acid (Xilong, Shenzhen, China); mobile phase B was acetonitrile (Xilong, Shenzhen, China) containing 0.05% formic acid; the gradient elution program was as follows: from 0 to 1 min, 10–20% B; from 1 to 9 min, 20–70% B; from 9 to 12.5 min, 70–95% B; from 12.5 to 13.5 min, 95% B; from 13.5 to 13.6 min, 95–10% B; from 13.6 to 16 min, 10% B. The flow rate was 0.35 mL/min; the column temperature was 40 °C; the injection volume was 2 μL. Concerning carotenoids, the UPLC conditions were as follows: the column was YMC C30 (3 μm, 100 mm × 2.0 mm i.d.); mobile phase A was a mixture of methanol and acetonitrile (1:3, *v*/*v*) containing 0.01% BHT and 0.1% formic acid; mobile phase B was methyl tert-butyl ether (MTBE) (Xilong, Shenzhen, China) containing 0.01% BHT; the gradient elution program was as follows: from 0 to 3 min, 0% B; from 3 to 5 min, 0–70% B; from 5 to 9 min, 70–95% B; from 10 to 11 min, 95–0% B. The flow rate was 0.8 mL/min; the column temperature was 28 °C; the injection volume was 2 μL.

All mass spectrometry analyses were conducted using the QTRAP^®^ 6500+ triple quadrupole-linear ion trap mass spectrometer (SCIEX, Shanghai, China). Flavonoids were analyzed via an electrospray ionization (ESI) source in the positive and negative ion switching mode. The ion source temperature was set at 550 °C, the spray voltage was 5500 V for positive ions and −4500 V for negative ions, the curtain gas (CUR) was maintained at 35 psi, and the scheduled MRM mode was employed. Carotenoids were analyzed using an atmospheric pressure chemical ionization (APCI) source in the positive ion mode. The ion source temperature was set at 350 °C, the curtain gas was maintained at 25 psi, and the scheduled MRM mode was utilized. Data acquisition was accomplished using Analyst 1.6.3 software, and quantitative analysis was carried out using MultiQuant 3.0.3 software.

Flavonoid metabolites were subjected to qualitative and quantitative analysis utilizing the MetWare self-built database (MWDB) and standards. In contrast, carotenoids were identified on the basis of the retention times and MS/MS fragmentation patterns of standards. Quality control (QC) samples were prepared by pooling equal quantities from each sample to evaluate the stability and reproducibility of the analytical system. Metabolite quantification data were processed through the imputation of missing values, normalization, and log-transformation, and subsequently, multivariate statistical analysis was conducted using R (v3.5.1). Principal component analysis (PCA) and orthogonal partial least squares discriminant analysis (OPLS-DA) were employed to visualize the overall differences between groups and to identify differentially abundant metabolites, respectively. Differentially abundant flavonoid metabolites were selected based on the criteria of VIP ≥ 1 (from the OPLS-DA model), |log_2_FC| ≥ 1, and *p* < 0.05; for carotenoids, the selection criteria were |log_2_FC| ≥ 1 and *p* < 0.05. Differentially abundant metabolites were annotated using the KEGG compound database and mapped to the KEGG pathway database, followed by pathway enrichment analysis using hypergeometric testing.

### 2.3. Transcriptome Sequencing and Data Analysis

Total RNA was extracted from 15 petal samples by using a total RNA extraction kit. The purity and concentration of the RNA were determined using a NanoDrop 2000 spectrophotometer (Thermo, Waltham, MA, USA), and RNA integrity was assessed on an Agilent 2100 Bioanalyzer (Agilent, Santa Clara, CA, USA) (RIN value ≥ 7.0). mRNA was enriched using Oligo(dT) magnetic beads, followed by fragmentation and the synthesis of first- and second-strand cDNA. The double-stranded cDNA was subjected to end repair, A-tailing, and adapter ligation, and subsequently amplified via PCR for library construction. The concentration of the library was accurately quantified using a Qubit 4.0 fluorometer, and the fragment size was analyzed with an Agilent 2100 Bioanalyzer. Libraries that passed the quality control were subjected to high-throughput sequencing on an Illumina NovaSeq 6000 platform (PE150 mode), yielding at least 6 Gb of clean data per sample, with a Q30 base percentage of not less than 96%.

Raw sequencing data were subjected to quality control using fastp (v0.19.5) to eliminate adapter sequences and low-quality reads (Q < 20), thereby yielding clean reads. Owing to the absence of a high-quality chromosome-level reference genome for *K. blossfeldiana*, de novo assembly of the clean reads was carried out using Trinity (v2.8.5) to generate Unigenes. The clean reads were mapped to the unigenes using RSEM (v1.3.1) in combination with Bowtie2 (v2.3.4.3), and FPKM values were computed for each gene as expression levels. Functional annotation of the Unigenes was accomplished through BLAST (v2.13.0) alignment against public databases such as Nr, Swiss-Prot, KEGG, GO, KOG/COG, and Pfam. DEGs were detected using DESeq2 (v1.22.1), with the selection criteria set at |log_2_FC| ≥ 1 and FDR < 0.05 (Benjamini–Hochberg correction). KEGG pathway enrichment and GO enrichment analyses were respectively conducted using hypergeometric tests and the R package clusterProfiler (v3.14.3).

### 2.4. Integrative Analysis of the Transcriptome and Metabolome

To conduct a systematic analysis of the associations between gene expression and metabolite accumulation, this study carried out an integrated analysis of metabolomics and transcriptomics. Initially, KEGG co-enrichment analysis was executed to identify the metabolic pathways that were significantly enriched (*p* < 0.05) by both DEGs and DAMs.

### 2.5. qRT-PCR Validation

To validate the reliability of RNA-seq data, six key genes associated with flower color formation (PAL, CHS, CHI, FLS, UFGT, and ZDS) were chosen for real-time quantitative PCR (qRT-PCR) analysis. The identical RNA batch utilized for transcriptome sequencing was employed, and first-strand cDNA was synthesized using a commercially available reverse transcription kit. Gene-specific primers were designed with Primer Premier 6.0 software, ensuring that the amplicon lengths were maintained within the range of 80 to 200 bp, and were synthesized by Bioengineering Co., Ltd. (Shanghai, China) (Table S2). Actin was employed as the reference gene (determined through prior screening for expression stability). The qRT-PCR reaction mixture was composed of ChamQ Universal SYBR qPCR Master Mix (Vazyme, Nanjing, China), and amplification was carried out on an ABI Prism 7500 real-time PCR system (Thermo, San Jose, CA, USA). Each sample was analyzed in three biological replicates and three technical replicates. Relative gene expression levels were calculated using the 2^−ΔΔCt^ method. One-way ANOVA and Duncan’s multiple range test were conducted using SPSS 26.0 software (*p* < 0.05).

### 2.6. Statistical Analysis

All experiments incorporated three independent biological replicates. Statistical analyses were performed using IBM SPSS Statistics (version 26). Group differences were assessed by one-way analysis of variance (ANOVA), and post-hoc pairwise comparisons were conducted using Duncan’s multiple range test when the ANOVA results indicated statistical significance. Data are presented as the mean ± standard deviation (SD), with error bars in all figures representing SD.

## 3. Results

### 3.1. Overview of Flavonoid and Carotenoid Metabolism Profiles

To conduct an in-depth analysis of the metabolic disparities in pigment metabolism across different colored *K. blossfeldiana* cultivars, this study utilized UPLC-MS/MS for targeted quantitative analysis of petal samples obtained from five cultivars (GLT, LD, LN, HHL, JBY). Quality control analysis demonstrated that the QC samples from each group exhibited a high degree of overlap on the total ion current chromatograms, featuring stable metabolite peak shapes and retention times, as depicted in Figure S1A,B. This indicates that the data quality is reliable and suitable for subsequent analysis. Furthermore, quantitative assessment of QC reproducibility showed that approximately 98% of the detected flavonoids and approximately 99% of the detected carotenoids had RSD (CV) values below 20%, and that nearly all detected metabolites (approaching 100% for both compound classes) had RSD values below 30%. These results further confirm the analytical reproducibility of both metabolomic datasets.

A total of 168 metabolites were identified within the flavonoid metabolome, encompassing 17 categories, namely flavonols (44), flavones (29), dihydroflavones (11), flavanols (9), dihydroflavonols (8), coumarins (8), phenolic acid derivatives (6), chalcones (4), flavonoid C-glycosides (4), isoflavones (3), proanthocyanidins (1), aurones (1), and tannins (1). Additionally, a total of 72 carotenoid metabolites were identified, consisting of 64 xanthophylls and 8 carotenes. The two categories of metabolites exhibited significant differentiation trends among cultivars, and the specific classification statistics are presented in Table 1.

**Figure 2:**
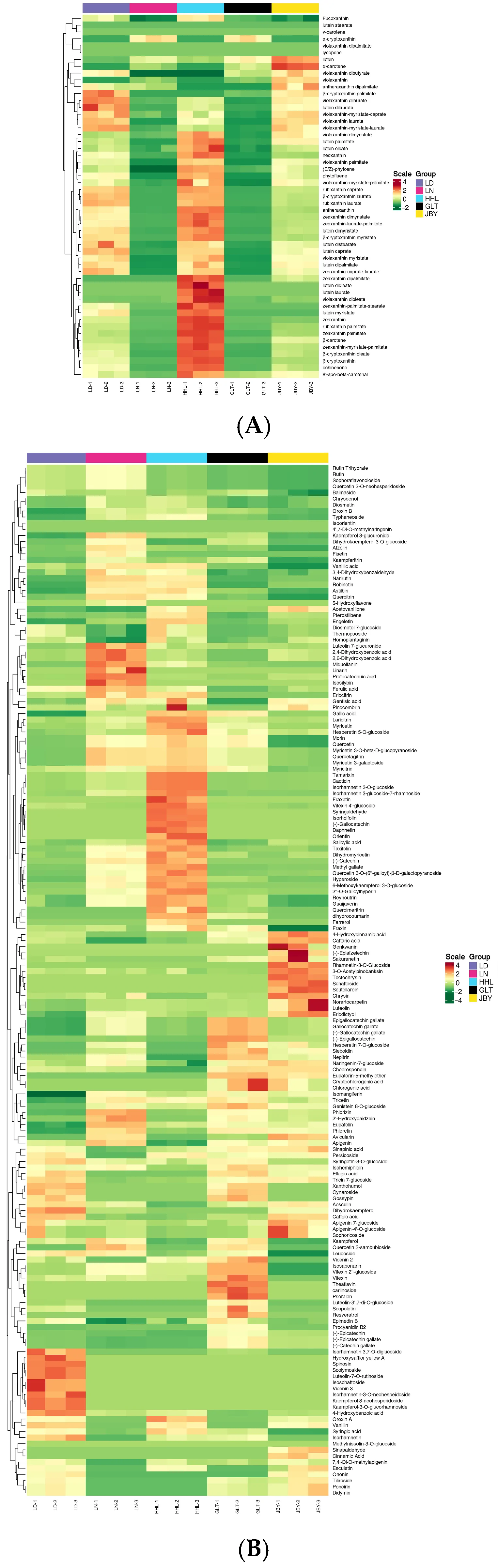
Cluster heatmap of metabolites in five different colored *K. blossfeldiana* cultivars. (**A**) Heatmap of carotenoid clustering analysis for five different colored *K. blossfeldiana* cultivars. (**B**) Heatmap of flavonoid clustering analysis for five different colored *K. blossfeldiana* cultivars. The horizontal axis denotes sample names, while the vertical axis denotes metabolite information. Different colors signify the varying values obtained subsequent to the normalization of content (red signifies high levels, and green signifies low levels).

The clustering heat map of the two categories of metabolites demonstrated distinct intra-group clustering among the cultivars at the metabolic level. The white cultivar GLT and the yellow cultivar LN showed highly similar carotenoid profiles, both displaying low carotenoid accumulation but elevated levels of flavonoids. Conversely, the red cultivar HHL, the purple–red cultivar LD, and the orange cultivar JBY clustered into another branch characterized by high carotenoid content; however, they exhibited divergence in flavonoid accumulation (HHL had higher levels, whereas LD and JBY had lower levels), which reflected the correspondence between flower color and metabolic profiles (Figure 2A,B).

### 3.2. Differential Metabolite Screening

White petals are generally regarded as the baseline state in pigment biosynthesis pathways, with their pigment metabolic network functioning at a low, basal level of activity. Consequently, this study concentrated on comparing differentially accumulated metabolites between GLT (featuring white petals) and other cultivars displaying distinct flower colors. The objective was to maximize the identification of specific accumulation patterns of pigments among colored cultivars (purple–red LD, yellow LN, red HHL, and orange JBY), thus offering crucial insights into the metabolic regulation underlying the transition from white to other flower colors. Based on the thresholds of VIP ≥ 1, FC ≥ 2 or ≤0.5, and *p* < 0.05, differentially accumulated metabolites were screened separately from the perspectives of flavonoids and carotenoids.

**Figure 3:**
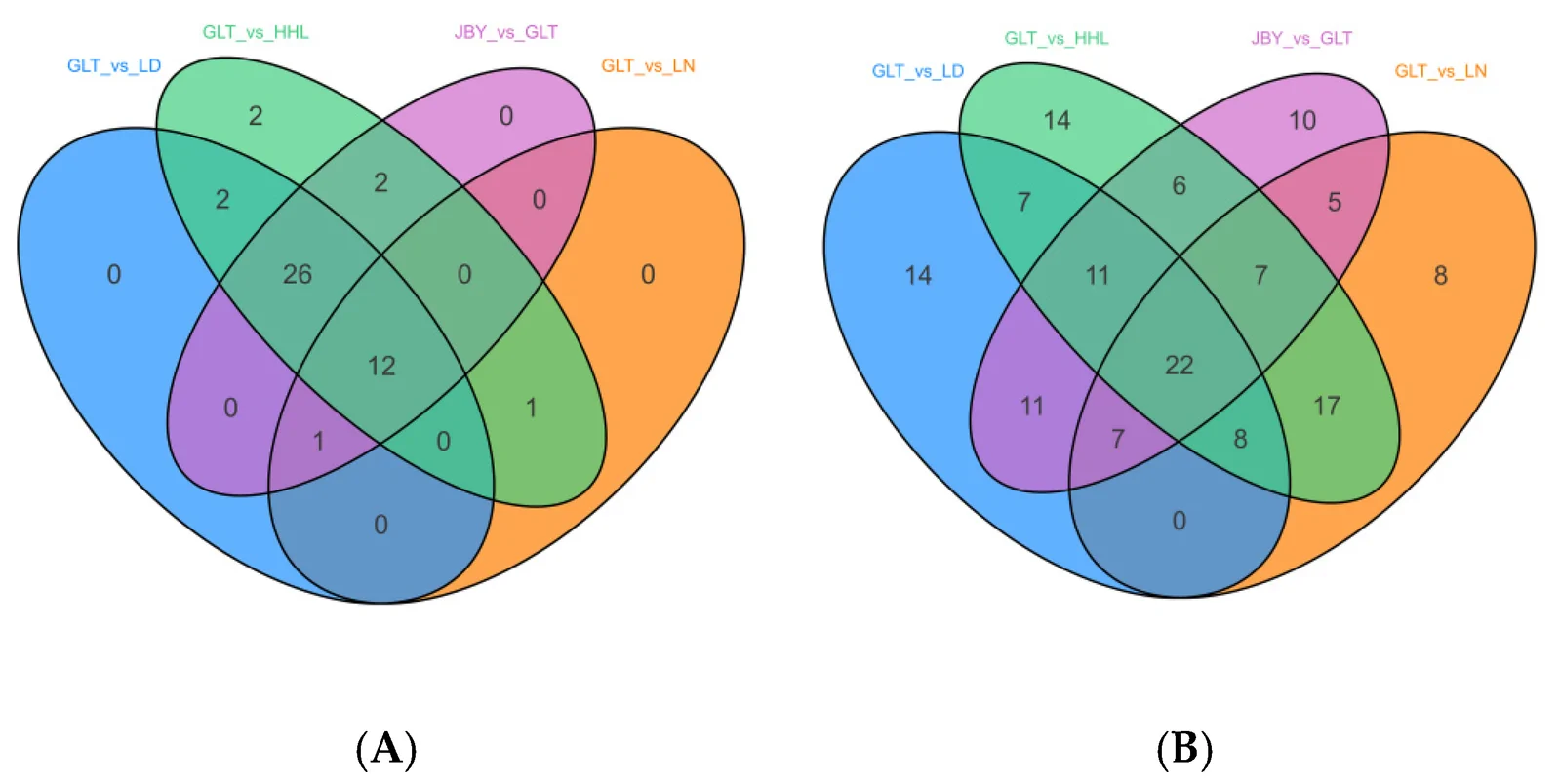
Metabolite analysis of the accumulated differences associated with pigment biosynthesis in *K. blossfeldiana* of different colors. (**A**) Venn diagram of carotenoid differential metabolites. (**B**) Venn diagram of differentially metabolized flavonoid compounds. Each circle in the figure represents a comparison group. The number in the overlapping area between circles denotes the quantity of differentially expressed metabolites shared among the groups, whereas the number in the non-overlapping regions represents the quantity of differentially expressed metabolites unique to each respective group.

Regarding carotenoid differential metabolites, 41, 14, and 45 differentially expressed carotenoid metabolites were identified in the comparisons of GLT vs. LD, GLT vs. LN, and GLT vs. HHL, respectively. In the comparison of GLT vs. JBY, 41 differentially expressed carotenoid metabolites were also found. The distribution of the differential profiles among the four groups was highly uneven. Specifically, the purple–red LD and red HHL exhibited a high overlap in their differential profiles relative to GLT (40 shared metabolites), whereas the yellow LN and orange JBY shared only 13 differential metabolites. This finding suggests that purple–red and red cultivars, as well as yellow and orange cultivars, follow similar regulatory mechanisms in carotenoid remodeling (Figure 3A). The results of the Venn diagram further indicated that 12 metabolites were commonly differentially expressed across the four comparisons, and 40 metabolites were shared between LD and HHL. This implies that purple-red and red cultivars share a carotenoid differentiation pattern centered on the accumulation of xanthophyll esters, which might serve as an important metabolic basis for the formation of non-white flower colors.

During the screening of differentially accumulated flavonoid compounds, 80, 74, and 92 differentially expressed flavonoid metabolites were respectively identified in the comparisons of GLT vs. LD, GLT vs. LN, and GLT vs. HHL, whereas 79 were detected in the comparison of GLT vs. JBY. A total of 22 common differentially expressed metabolites were shared among all four comparisons, and an additional 46 metabolites were detected solely in single comparisons, which suggests that flavonoid differentiation among cultivars involves both shared patterns and cultivar-specific characteristics (Figure 3B). Venn analysis further demonstrated that the 22 metabolites common to all four groups may represent core flavonoid regulatory nodes underlying the transition from white petals to other petal colors, covering multiple classes such as flavonols, flavones, phenolic acid derivatives, and coumarins.

### 3.3. Metabolic Pathway Enrichment Analysis

The screening of the aforementioned differential metabolites demonstrated pigment differentiation characteristics among cultivars at the metabolite level. To further clarify the underlying regulatory mechanisms from the perspective of metabolic pathways, KEGG pathway enrichment analysis was conducted on the differentially expressed metabolites in the four comparisons.

**Figure 4:**
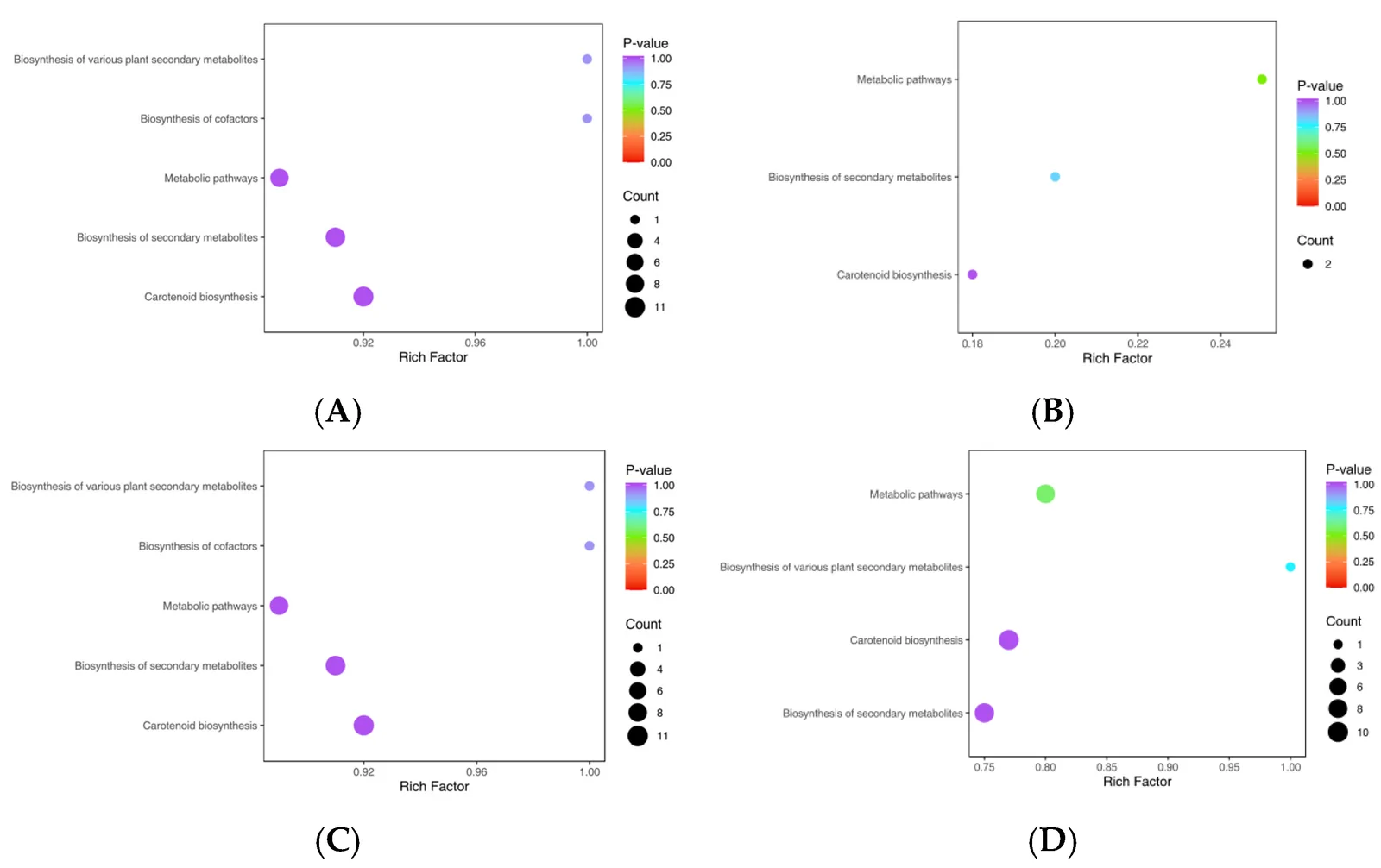
KEGG enrichment analysis of differentially accumulated carotenoid metabolites in *K. blossfeldiana* with distinct color cultivars. (**A**) GLT vs. LD, (**B**) GLT vs. LN, (**C**) GLT vs. HHL, (**D**) GLT vs. JBY. The *x*-axis represents the rich factor for each pathway, while the *y*-axis displays the pathway names. The color of the points denotes the *p*-value, where a redder color implies a more significant enrichment. The size of the points reflects the quantity of differentially expressed metabolites enriched in each pathway.

For carotenoids, the KEGG enrichment bubble plot indicated that the differentially expressed metabolites in the four comparisons were mainly enriched in pathways including carotenoid biosynthesis, biosynthesis of secondary metabolites, metabolic pathways, biosynthesis of various plant secondary metabolites, and biosynthesis of cofactors (Figure 4A–D). In the comparison group of GLT vs. LD (Figure 4A), differentially expressed metabolites were co-annotated to five KEGG pathways. Among these pathways, the *p*-values of differentially expressed metabolites in the three core pathways, namely, carotenoid biosynthesis, biosynthesis of secondary metabolites, and metabolic pathways, were comparable. This indicates that carotenoid-related differential metabolites are widely distributed both within the overall metabolic network and in carotenoid-specific biosynthetic pathways between white and purple–red petals. Moreover, although the Rich Factor values for the biosynthesis of various plant secondary metabolites and biosynthesis of cofactors pathways were approximately 1.0, the number of differentially expressed metabolites in these two pathways was relatively small, suggesting a limited quantity of such metabolites in these pathways. The comparison group of GLT vs. LN (Figure 4B) was annotated to only three pathways, with a relatively small number of differentially expressed metabolites in each pathway. The Rich Factor for carotenoid biosynthesis was approximately 0.18, and that for metabolic pathways was about 0.25, with *p*-values of approximately 0.9 and 0.5, respectively. These enrichment results suggest that although carotenoid-related differential metabolites were detected between white and yellow petals, their distribution across KEGG-annotated pathways is dispersed, indicating limited enrichment in the specific carotenoid biosynthetic pathway. The comparison group of GLT vs. HHL (Figure 4C) was jointly annotated to five pathways, and its enrichment patterns were highly similar to those of GLT vs. LD. Among these pathways, the counts for carotenoid biosynthesis, biosynthesis of secondary metabolites, and metabolic pathways were approximately 6 each. Their Rich Factors were about 0.92, 0.91, and 0.8, respectively, and the *p*-values were also close. These results suggest that carotenoid-related differential metabolites between white and red petals are similarly involved in core pathways such as carotenoid biosynthesis and secondary metabolite synthesis. The comparison group between GLT and JBY (Figure 4D) was jointly annotated to four pathways, among which carotenoid biosynthesis, biosynthesis of secondary metabolites, and metabolic pathways were the most significantly enriched. When compared with the LD and HHL groups, the enrichment factor of the carotenoid biosynthesis pathway in the JBY group exhibited a slight decrease, while the absolute number of differentially expressed metabolites in this pathway remained largely stable. Notably, the pathway of biosynthesis of various plant secondary metabolites also emerged in this group, but with a lower count value, suggesting fewer differentially expressed metabolites were enriched in this pathway. Overall, the carotenoid biosynthesis pathway was consistently annotated in all three comparisons, namely GLT vs. LD, GLT vs. HHL, and GLT vs. JBY, with a relatively high quantity of differentially expressed metabolites and a rich factor. Conversely, in the GLT vs. LN group (Figure 4B), the count for the carotenoid biosynthesis pathway was merely approximately 2, with a rich factor as low as 0.18, indicating notably weaker enrichment signals compared to other colored groups. Although none of the *p*-values attained the conventional statistical significance threshold (*p* < 0.05), considering the central role of carotenoid metabolites in petal pigmentation, the carotenoid biosynthesis pathway is still identified as a key candidate pathway for further exploration. Moreover, the metabolic pathways and the biosynthesis of secondary metabolites were consistently detected across multiple comparisons, suggesting that the differences in carotenoids between colored and white petals are not isolated occurrences but are instead embedded within a broader network of secondary metabolic regulation.

**Figure 5:**
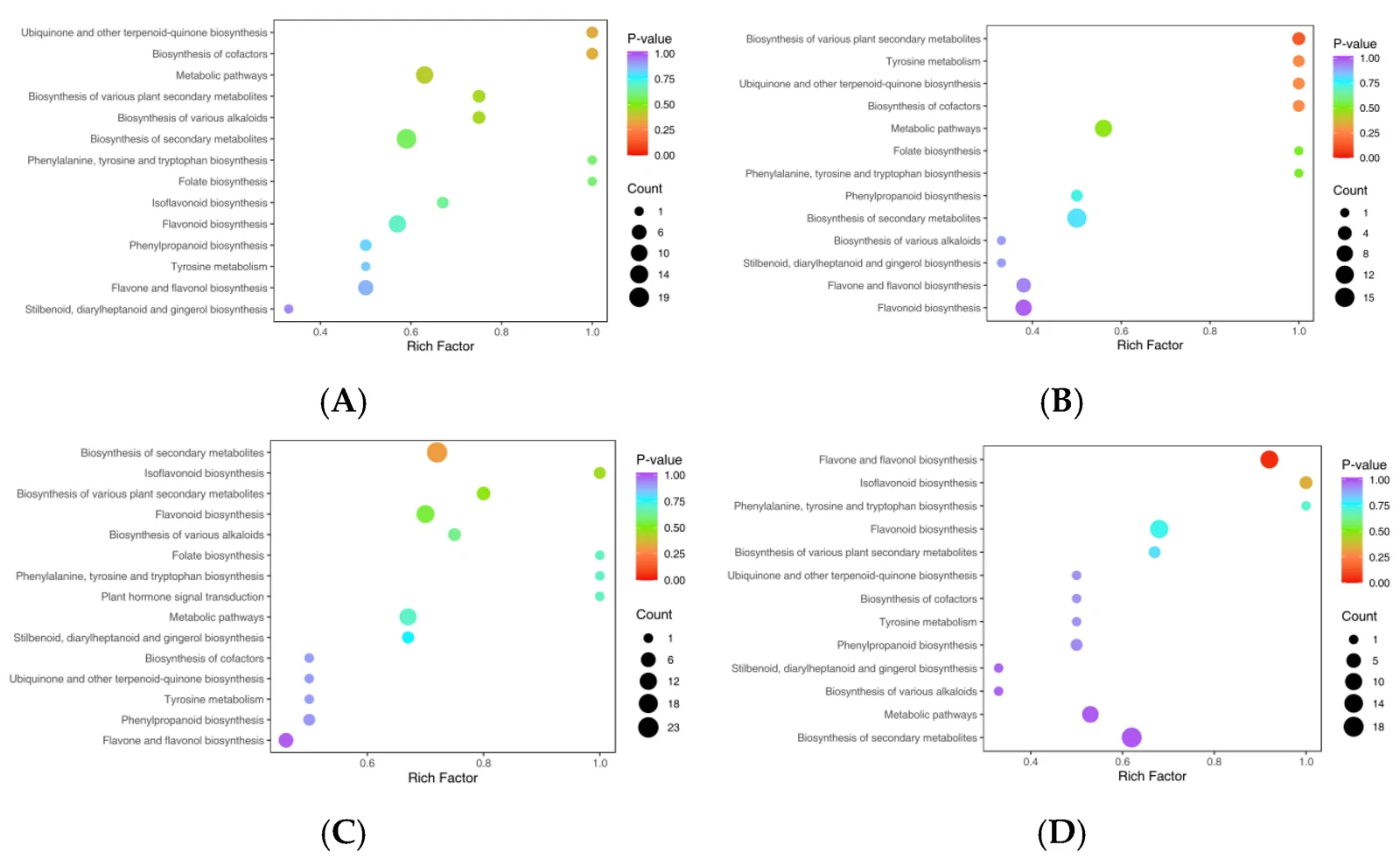
KEGG enrichment analysis of differentially accumulated flavonoid metabolites in *K. blossfeldiana* with distinct color cultivars. (**A**) GLT vs. LD, (**B**) GLT vs. LN, (**C**) GLT vs. HHL, (**D**) GLT vs. JBY. The *x*-axis represents the rich factor for each pathway, while the *y*-axis displays the pathway names. The color of the points denotes the *p*-value, where a redder color implies a more significant enrichment. The size of the points reflects the quantity of differentially expressed metabolites enriched in each pathway.

For flavonoids, the KEGG enrichment bubble plot indicated that the differentially expressed metabolites across the four comparisons were predominantly enriched in pathways including flavonoid biosynthesis, flavone and flavonol biosynthesis, phenylpropanoid biosynthesis, biosynthesis of secondary metabolites, and metabolic pathways (Figure 5A–D). In the GLT and LD groups (Figure 5A), a substantial quantity of differentially expressed metabolites were detected in secondary metabolite biosynthesis, flavonoid biosynthesis, and metabolic pathways. The entire phenylpropanoid–flavonoid metabolic axis was notably enriched, spanning from upstream phenylalanine biosynthesis to downstream flavonoid and flavonol biosynthesis. This indicates that, in comparison to white petals, purple–red petals experienced a comprehensive cascade activation of the flavonoid synthesis pathway. The overall number of differentially expressed metabolites in the GLT vs. LN group (Figure 5B) was relatively low. Although differences were detected in the upstream Phenylalanine and Phenylpropanoid pathways, the quantity of such metabolites was limited. In contrast, the downstream flavonoid biosynthesis and flavone and flavonol biosynthesis pathways exhibited a relatively higher count of differentially expressed metabolites. This suggests that the metabolic differences between yellow and white petals are mainly concentrated at the downstream stage. The comparison between the GLT and HHL groups (Figure 5C) demonstrated the most comprehensive secondary metabolic reprogramming characteristics. The quantity of differentially metabolized compounds in the secondary metabolite biosynthesis pathway was significantly greater than that in flavonoid biosynthesis, and pathways related to stilbenoid, isoflavonoid, and alkaloid synthesis were also notably annotated. This suggests that the disparities between red and white petals are not confined to flavonoid metabolism solely but encompass coordinated alterations throughout the entire phenylpropanoid-derived polyphenol-secondary metabolic network. The quantity of differentially accumulated metabolites in the flavonoid biosynthesis pathway between the GLT and JBY groups (Figure 5D) was also comparatively high. In contrast, pathways such as flavone and flavonol biosynthesis and isoflavonoid biosynthesis demonstrated very few absolute disparities in metabolite levels, yet they displayed highly specific enrichment signals (Rich Factor close to 1.0). This indicates that alterations in flavonoid metabolism in orange petals are not merely attributable to overall increases or decreases; instead, they involve precise differential regulation of downstream branch pathways. Overall, the four comparisons unveiled four distinct strategies in flavonoid metabolism across different petal colors of *K. blossfeldiana*. The comparison between the GLT and LD groups showed a comprehensive cascade activation of the core flavonoid pathways. The comparison between the GLT and LN groups exhibited minor disparities in the upstream precursor metabolism but significant enrichment in the downstream flavonoid synthesis pathways. The comparison between the GLT and HHL groups demonstrated an extensive reprogramming of the secondary metabolic networks, where flavonoids accounted for only a part of the broader polyphenol metabolic changes. The comparison between the GLT and JBY groups displayed both an increase in the total flavonoid levels and a highly specific regulation of the downstream branch pathways. These results suggest that the differences in flavonoid metabolism among colored petals are not regulated by a single pattern but rather involve a multidimensional differential regulation that encompasses the scope of metabolic networks, the hierarchical integrity of pathways, and branch specificity.

### 3.4. Transcriptome Sequencing and Differential Gene Expression Analysis

To elucidate the molecular mechanisms underlying petal pigment differentiation in distinct flower-color cultivars of *K. blossfeldiana*, this research conducted transcriptome sequencing on 15 petal samples (with 3 biological replicates each) sourced from 5 flower-color cultivars. Subsequent to quality control filtering, a total of approximately 135.5 Gb of clean data were acquired, with an average of 9.03 Gb per sample. The Q30 percentage fell within the range of 96.32% to 97.10%, and the error rate was 0.02% for all samples, suggesting that the sequencing data were reliable and appropriate for subsequent analysis (Table S1). The GC content was approximately 48%, which was in line with the typical characteristics of plant transcriptomes. Employing Trinity for de novo assembly, a total of 275,516 transcripts and 179,955 unigenes were acquired, with average lengths of 1160 bp and 1410 bp, respectively. The N50 values were 1861 bp and 2053 bp, whereas the N90 values were 489 bp and 646 bp, which suggests a high-quality assembly (Figure S2A). Functional annotation demonstrated that 142,262 unigenes (79.05%) were annotated in at least one public database, with annotation rates of 72.27% in Nr, 55.89% in KEGG, 56.01% in Swiss-Prot, 67.23% in GO, 44.74% in KOG, and 47.08% in Pfam (Figure S2B). PCA conducted on FPKM values effectively classified the five cultivars into distinct clusters. The first two principal components accounted for the majority of the variation (Figure S2C). Significantly, the white cultivar GLT and the yellow cultivar LN were in relatively close proximity in the PCA plot, whereas the red cultivars HHL and LD formed a separate branch. This clustering pattern was in line with the metabolic profile differentiation identified in the metabolomics analysis.

**Figure 6:**
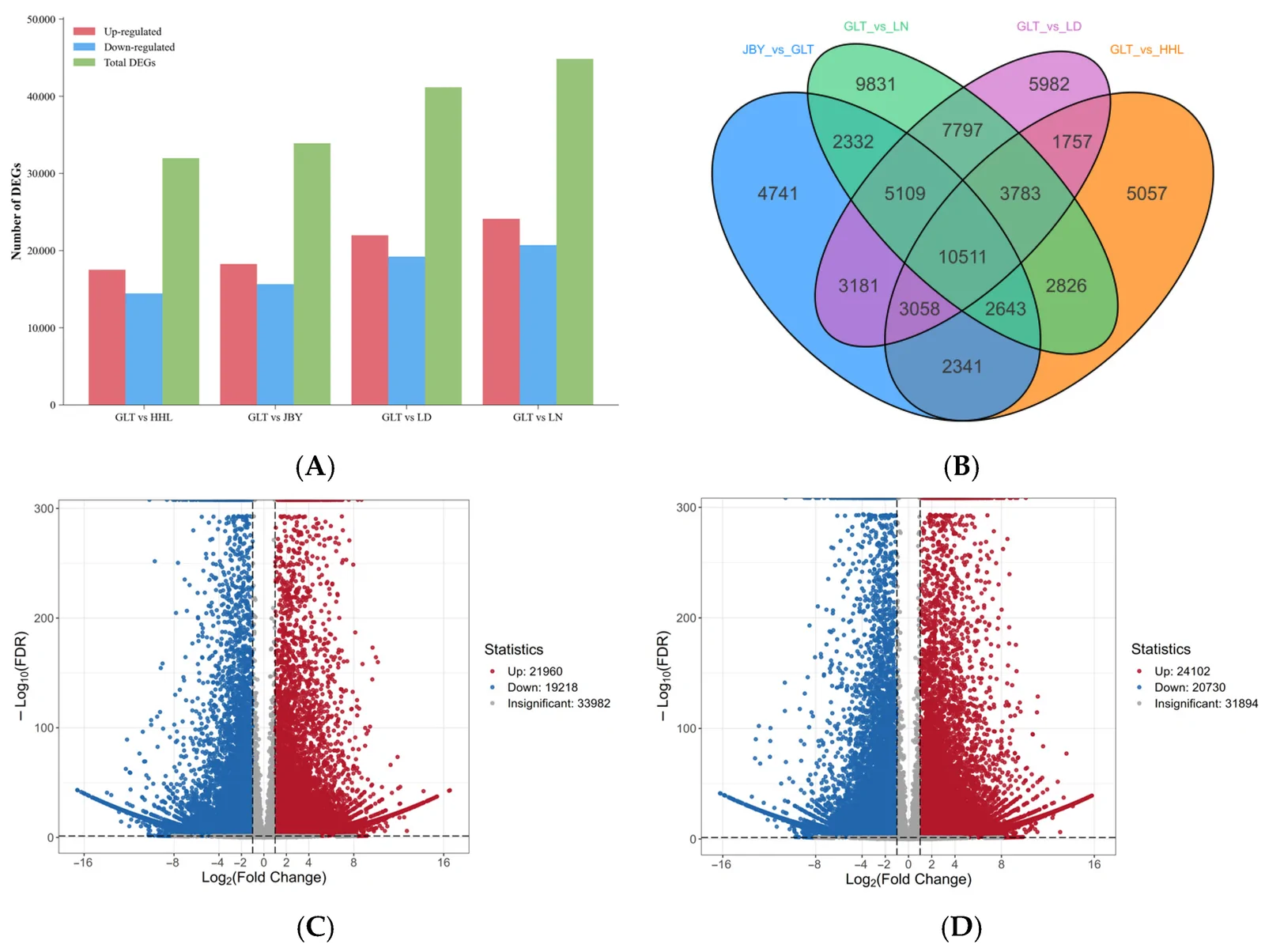

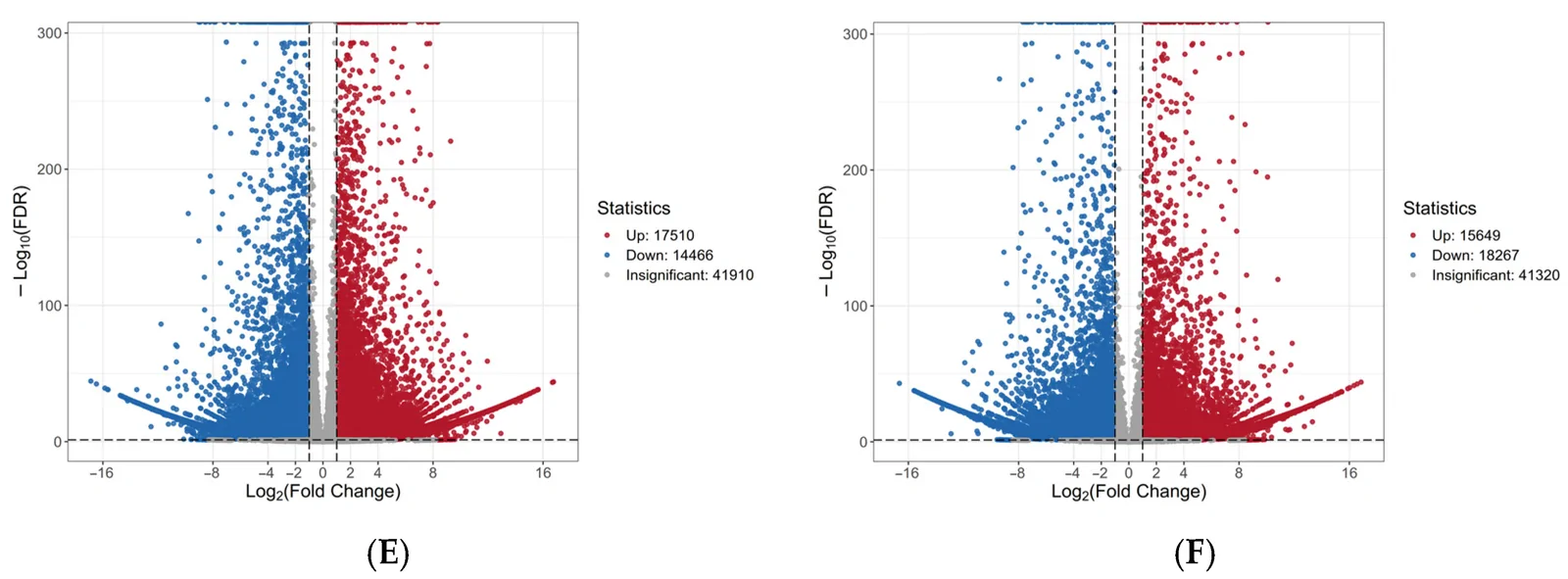
Overview of DEGs across 4 color variants of *K. blossfeldiana*. (**A**) Statistics of up- and down-regulated DEGs in 4 pairwise comparisons. (**B**) Venn diagram showing the overlap of DEGs among GLT vs. LN, GLT vs. LD, GLT vs. JBY, and GLT vs. HHL. (**C**) Volcano plot of DEGs in GLT vs. LD. (**D**) Volcano plot of DEGs in GLT vs. HHL. (**E**) Volcano plot of DEGs in GLT vs. JBY. (**F**) Volcano plot of DEGs in GLT vs. LN.

Differential expression analysis was conducted using DESeq2, with |log_2_FC| ≥ 1 and FDR < 0.05 serving as the selection criteria. Taking the white cultivar GLT as the control, a substantial number of DEGs were identified in all four comparison groups. Specifically, the comparison of GLT vs. LD resulted in 41,178 DEGs (21,960 up-regulated and 19,218 down-regulated), GLT vs. LN yielded 44,832 DEGs (24,102 up-regulated and 20,730 down-regulated), GLT vs. HHL produced 31,976 DEGs (17,510 up-regulated and 14,466 down-regulated), and GLT vs. JBY generated 33,916 DEGs (18,267 up-regulated and 15,649 down-regulated) (Figure 6A). The GLT vs. LN comparison group exhibited the highest quantity of DEGs, suggesting the most extensive transcriptome reprogramming between white and yellow petals. Venn diagram analysis revealed a shared core set of DEGs across the four comparisons, along with DEGs unique to each comparison group, implying that the divergence among different flower color cultivars encompasses both common regulatory mechanisms and cultivar-specific processes (Figure 6B). The volcano plot visually depicted the distribution characteristics of DEGs across the four comparison groups within a two-dimensional space defined by the magnitude of expression change and statistical significance. In the comparison between the GLT group and the LD group, the DEGs exhibited a significant bidirectional symmetric distribution, with a substantial number of genes clustered in regions of high significance. This suggested that the transition from white to purple–red flower color entails extensive and robust bidirectional transcriptional regulation (Figure 6C). The comparison between the GLT group and the LN group demonstrated the broadest scatter distribution, with some genes attaining the highest levels of expression difference. This indicates that the formation of yellow petals may be accompanied by the most dramatic transcriptome remodeling (Figure 6D). In contrast, the distribution of DEGs between the GLT group and the HHL group is relatively concentrated, with fewer genes displaying extreme expression differences. This reflects relatively limited transcriptional reprogramming between red and white cultivars (Figure 6E). In the comparison between the GLT group and the JBY group, the DEGs are primarily concentrated within moderate ranges of expression change. This indicates that the differences in white versus orange flower color mainly involve moderate adjustments in gene expression.

**Figure 7:**
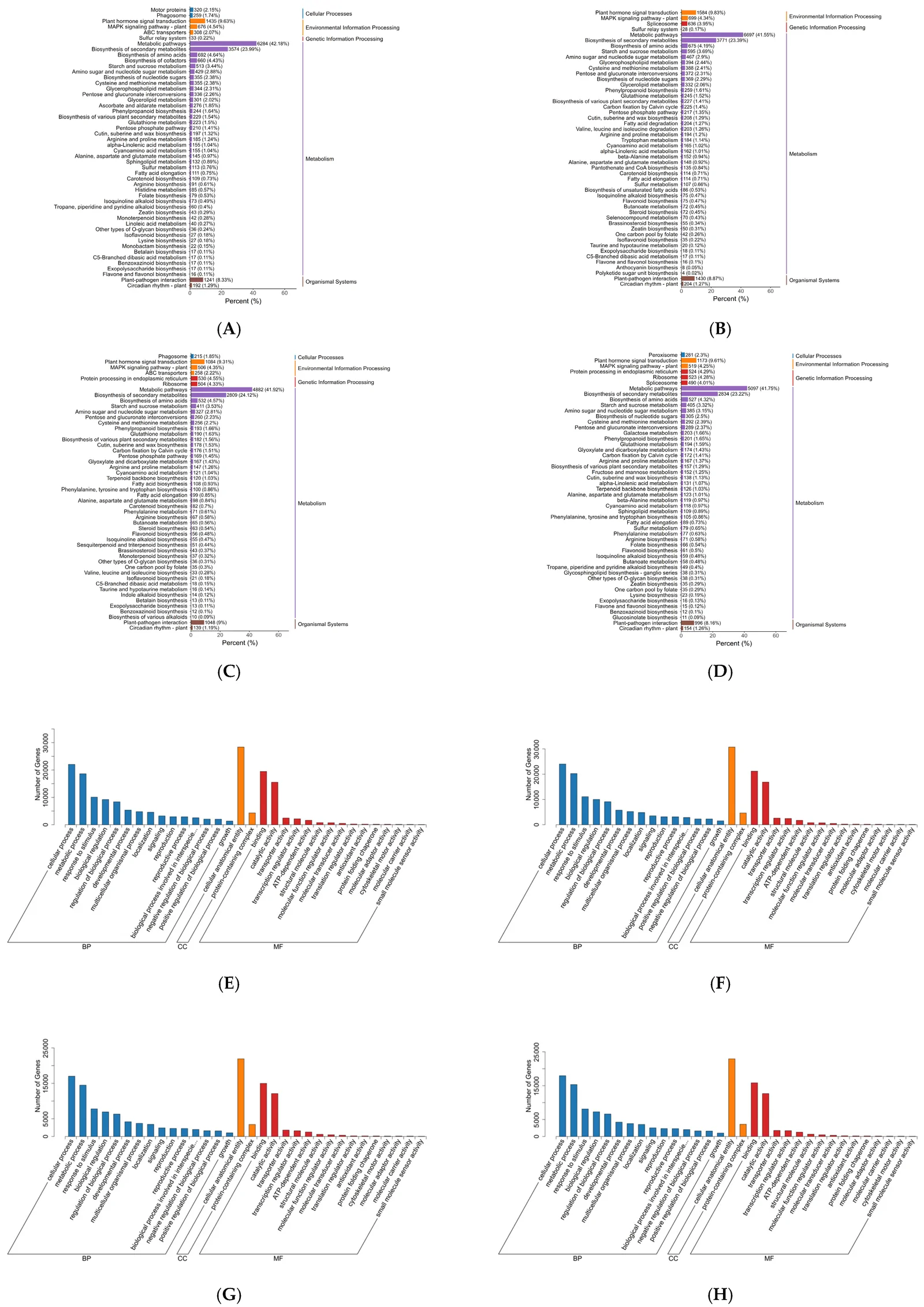
KEGG and GO pathway enrichment analysis of differentially expressed genes among 5 cultivars of *K. blossfeldiana* with distinct flower colors. (**A**) KEGG enrichment analysis of DEGs in GLT vs. LD. (**B**) KEGG enrichment analysis of DEGs in GLT vs. LN. (**C**) KEGG enrichment analysis of DEGs in GLT vs. HHL. (**D**) KEGG enrichment analysis of DEGs in GLT vs. JBY. (**E**) GO enrichment analysis of DEGs in GLT vs. LD. (**F**) GO enrichment analysis of DEGs in GLT vs. LN. (**G**) GO enrichment analysis of DEGs in GLT vs. HHL. (**H**) GO enrichment analysis of DEGs in GLT vs. JBY.

GO and KEGG enrichment analyses were conducted on the differentially expressed genes between the GLT and four colored cultivars (LD, LN, HHL, JBY). The findings indicated both commonalities and color-specific characteristics in the functional pathways across the comparison groups. The KEGG analysis demonstrated that all four groups were significantly enriched in pathways such as plant hormone signal transduction, plant–pathogen interaction, secondary metabolite biosynthesis, and phenylpropanoid biosynthesis. This suggests that the molecular basis of color differentiation encompasses extensive signaling and defense response networks. In the comparison of GLT and LD, the flavonoid and flavonol biosynthesis pathways were significantly enriched, which suggested that the accumulation of flavonoid metabolites might be associated with the purple–red coloration (Figure 7A). When comparing GLT with LN, apart from the flavonoid pathway, anthocyanin biosynthesis was also significantly enriched, implying active transcriptional regulation of the anthocyanin metabolic pathway in yellow cultivars (Figure 7B). In the comparison between GLT and HHL, pathways such as fatty acid elongation and terpenoid backbone biosynthesis were prominent, potentially linked to carotenoid and lipid-soluble pigment metabolism in red cultivars (Figure 7C). In the comparison of JBY and GLT, amino acid and sugar metabolism pathways, including alanine, aspartate, and glutamate metabolism, and nucleotide sugar metabolism, were significantly enriched, along with a continued significant enrichment in the flavonoid biosynthesis pathway. This suggested that orange color formation may involve the coordinated remodeling of nitrogen and carbon metabolism (Figure 7D). GO enrichment analysis demonstrated that all four groups exhibited significant enrichment in fatty acid derivative metabolism/biosynthesis and wax metabolism/biosynthesis at the biological process (BP) level. This finding suggests that cuticular wax and lipid metabolism are generally regulated during transcriptional differentiation among different flower color cultivars. At the molecular function (MF) level, oxidoreductase activity, transmembrane receptor protein kinase activity, and fatty acyl-CoA reductase activity were significantly enriched across all groups, which further corroborates the central role of lipid metabolism and signal perception in flower color variation.

### 3.5. Multi-Omics Integration: Metabolite–Gene Associations and Network Analysis

**Figure 8:**
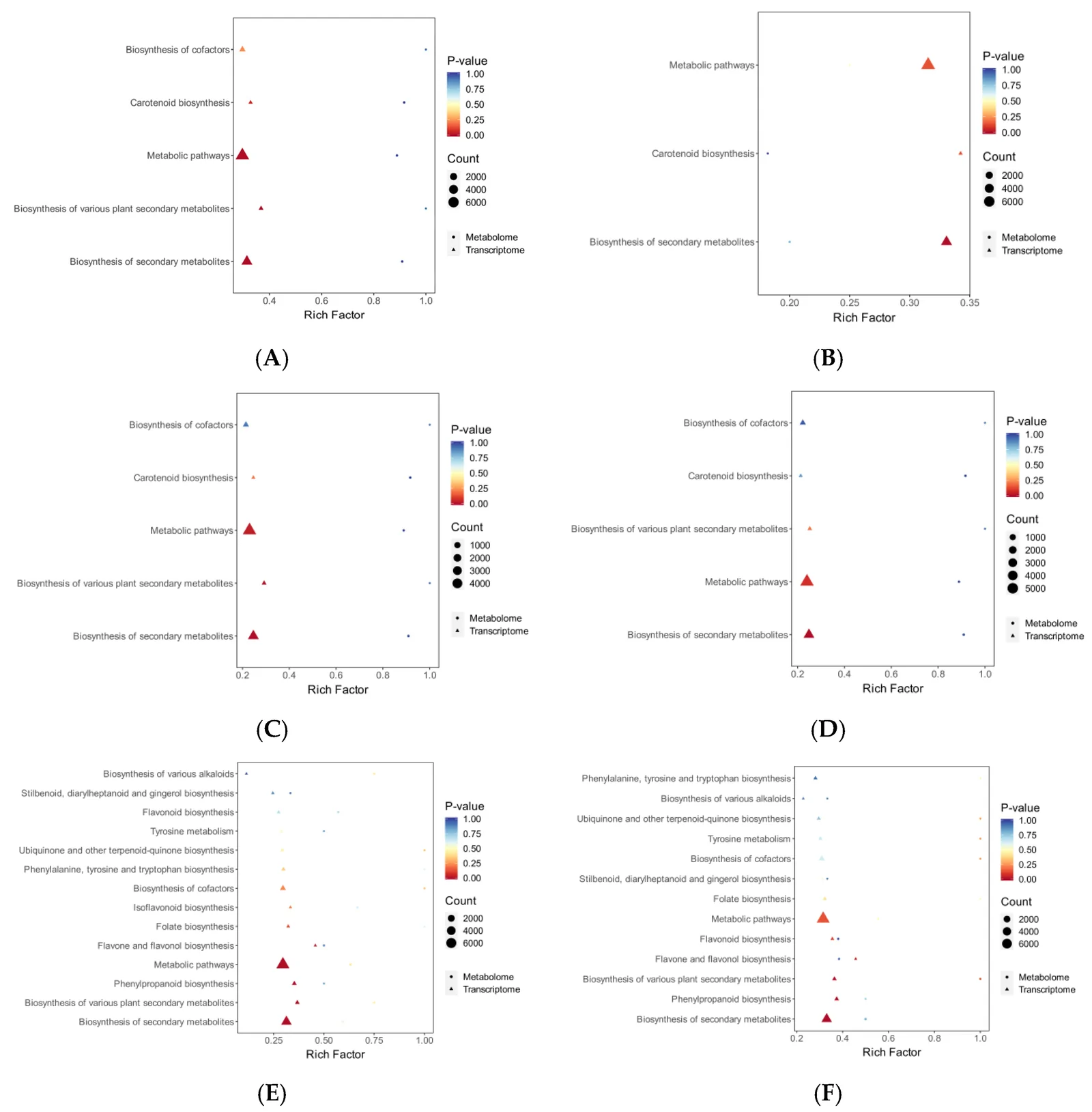

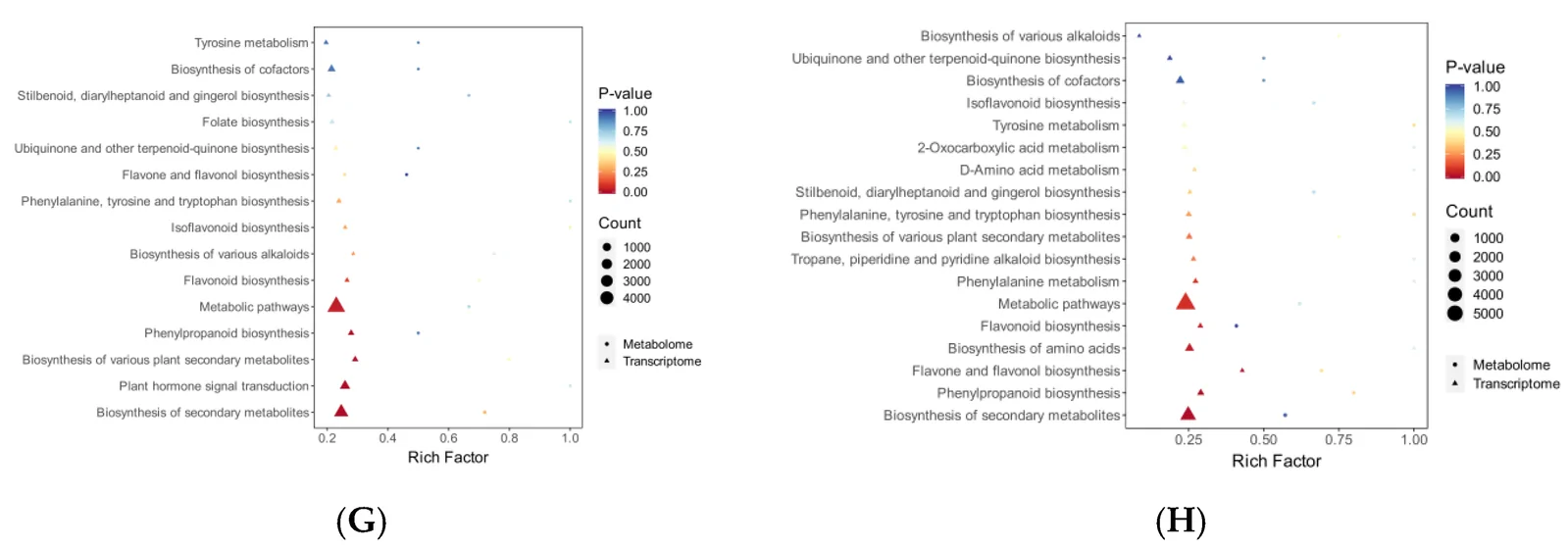
Combined KEGG enrichment bubble plots of carotenoid and flavonoid metabolomes with transcriptomes are presented. (**A**) A combined KEGG enrichment bubble plot of the carotenoid metabolome and transcriptome in the comparison between GLT and LD. (**B**) A combined KEGG enrichment bubble plot of the carotenoid metabolome and transcriptome in the comparison between GLT and LN. (**C**) A combined KEGG enrichment bubble plot of the carotenoid metabolome and transcriptome in the comparison between GLT and HHL. (**D**) A combined KEGG enrichment bubble plot of the carotenoid metabolome and transcriptome in the comparison between GLT and JBY. (**E**) A combined KEGG enrichment bubble plot of the flavonoid metabolome and transcriptome in the comparison between GLT and LD. (**F**) A combined KEGG enrichment bubble plot of the flavonoid metabolome and transcriptome in the comparison between GLT and LN. (**G**) A combined KEGG enrichment bubble plot of the flavonoid metabolome and transcriptome in the comparison between GLT and HHL. (**H**) A combined KEGG enrichment bubble plot of the flavonoid metabolome and transcriptome in the comparison between GLT and JBY. The *x*-axis denotes the enrichment factor of the pathway across diverse omics, whereas the *y*-axis signifies the KEGG pathway name. The gradient from red to yellow to blue reflects the significance of enrichment, spanning from high to medium to low, as denoted by the *p*-value. The shape of the bubbles represents distinct omics types, and the size of the bubbles corresponds to the quantity of differentially expressed metabolites or genes; larger quantities lead to larger dots.

To conduct an in-depth analysis of the mechanism underlying flower color diversification at the systems level, we carried out comprehensive transcriptome and metabolome analyses of four additional cultivars, with GLT serving as the control. The integrated KEGG pathway enrichment analysis indicated that secondary metabolite biosynthesis, phenylpropanoid biosynthesis, flavonoid biosynthesis, flavonol biosynthesis, and carotenoid biosynthesis were commonly detected across GLT and the four comparison groups. This finding suggests that these pigment metabolic pathways constitute the core framework that drives the differences in flower color among various *K. blossfeldiana* cultivars (Figure 8).

**Figure 9:**
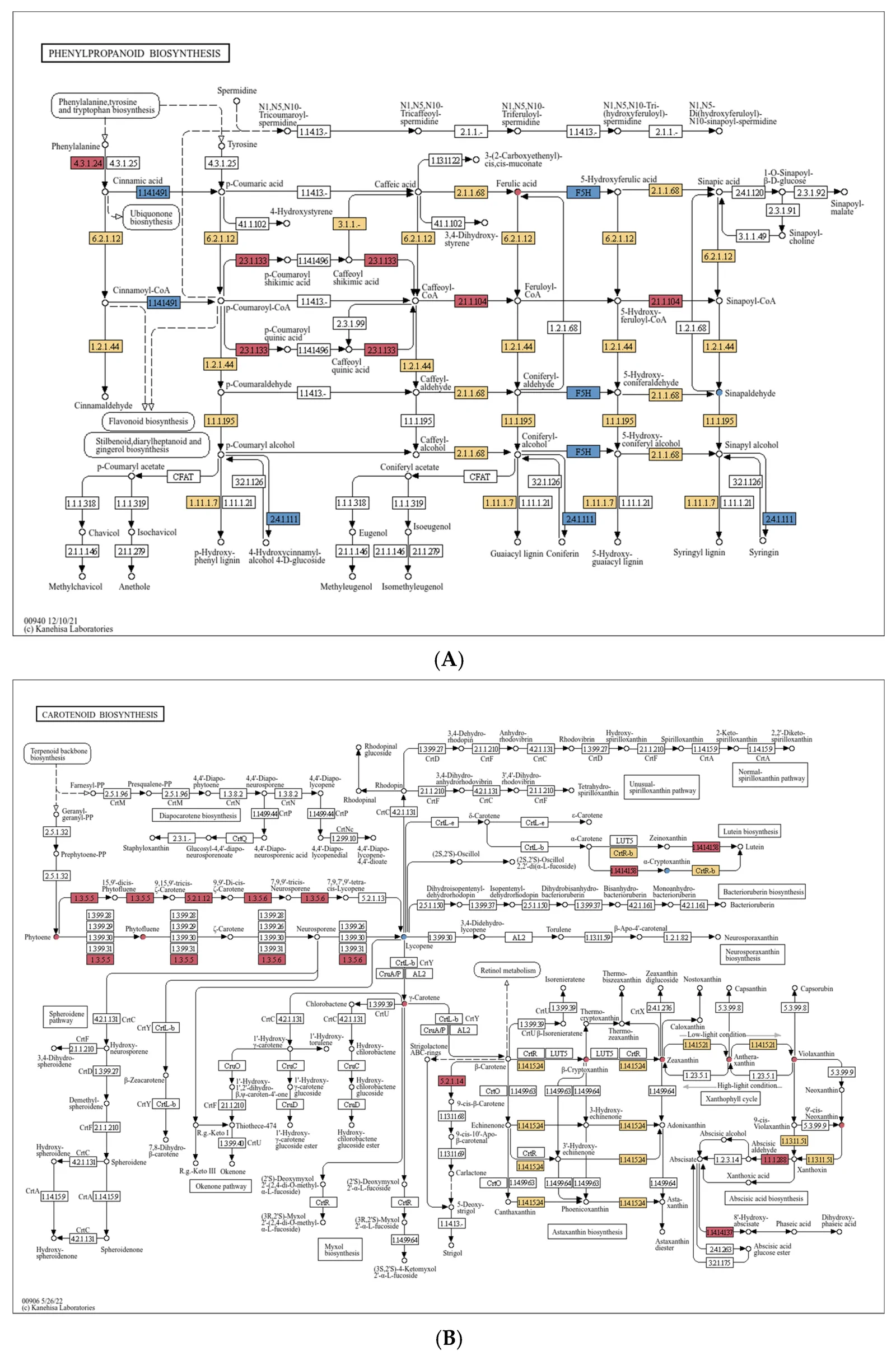
Integrated KEGG pathways of phenylpropanoid–flavonoid and carotenoid biosynthesis. (**A**) Phenylpropanoid and flavonoid biosynthesis pathway. (**B**) Carotenoid biosynthesis pathway. PAL, CHS, and CHI form the common upstream module; FLS and UFGT were located at the downstream branch points directing metabolite flux toward flavonols and anthocyanins, respectively. ZDS catalyzes the desaturation step that extends the conjugated double-bond system, a key determinant of carotenoid color.

Based on this, we conducted a more in-depth focus on the structural genes of two pigment biosynthetic pathways. The phenylpropanoid biosynthesis pathway functions as the upstream entry point for flavonoid synthesis. Genes in this pathway encode PAL, which facilitates the conversion of phenylalanine to cinnamic acid, thereby supplying precursors for subsequent flavonoid synthesis. CHS and CHI are tasked with condensing 4-coumaroyl-CoA with malonyl-CoA to form naringenin chalcone, which is then isomerized into naringenin—a crucial step in establishing the flavonoid backbone. FLS catalyzes the transformation of dihydroflavonols to flavonols, which determines the accumulation of flavonol pigments. UFGT catalyzes the glycosylation of anthocyanidin aglycones, which has an impact on pigment stability and water solubility. In the carotenoid biosynthesis pathway, ZDS catalyzes the multi-step dehydrogenation of ζ-carotene to lycopene. It is a critical enzyme for carotenoid chain elongation and the formation of conjugated double-bond systems, directly influencing the composition and intensity of yellow pigments in petals (Figure 9).

Based on the above results, combined with previous metabolomics analysis and differential expression data, we hypothesize that six key structural genes—PAL, CHS, CHI, FLS, and UFGT in the phenylpropanoid pathway, and ZDS in the carotenoid pathway—play potential regulatory roles in color formation by exhibiting distinct expression patterns across different flower-colored cultivars. These genes are enriched in the phenylpropanoid and carotenoid metabolic pathways, respectively, and show significant expression differences between colored and white cultivars, suggesting they are core candidate genes regulating *K. blossfeldiana* flower coloration.

### 3.6. WGCNA Reveals Gene Modules Associated with Pigment Metabolism

**Figure 10:**
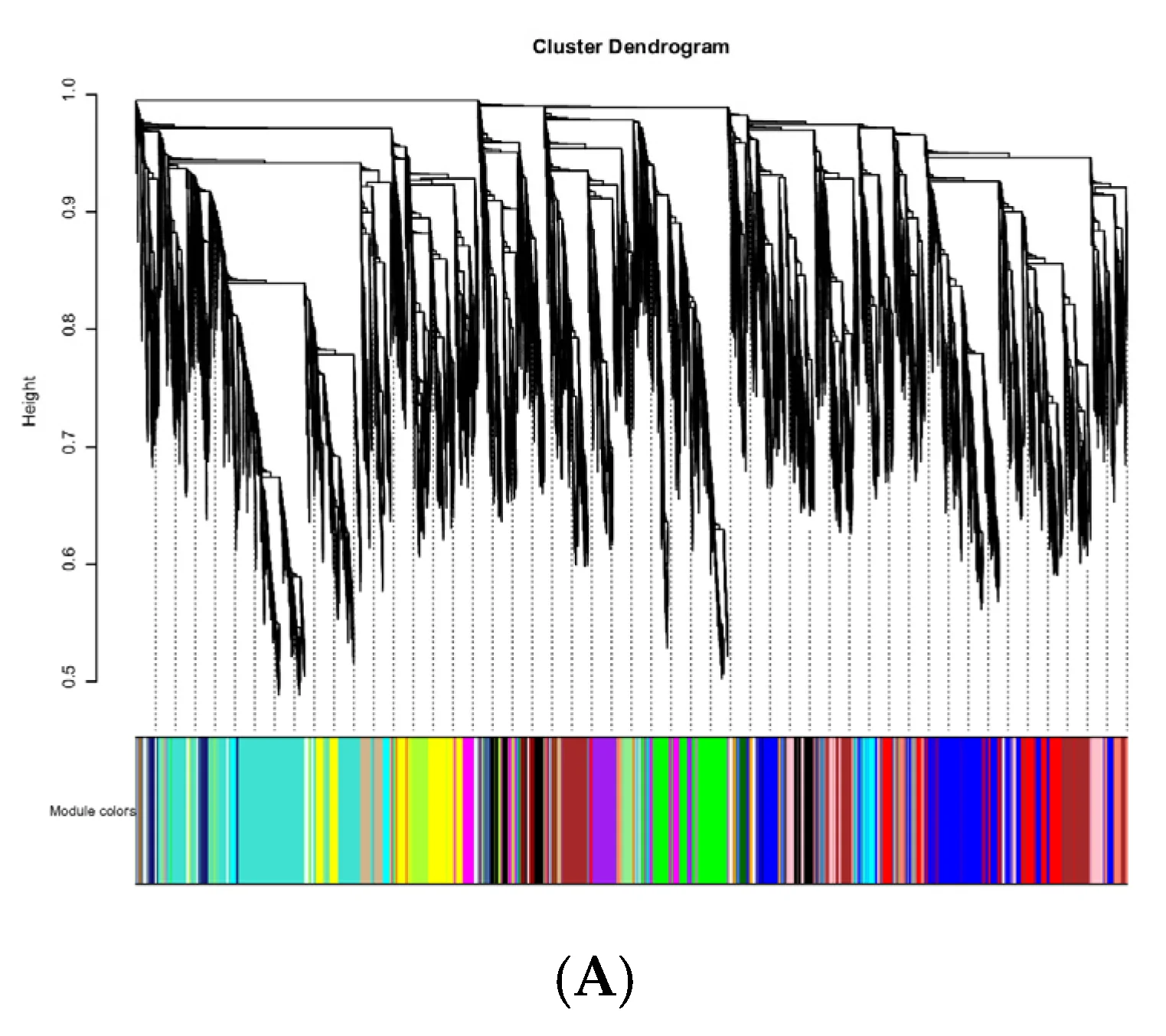

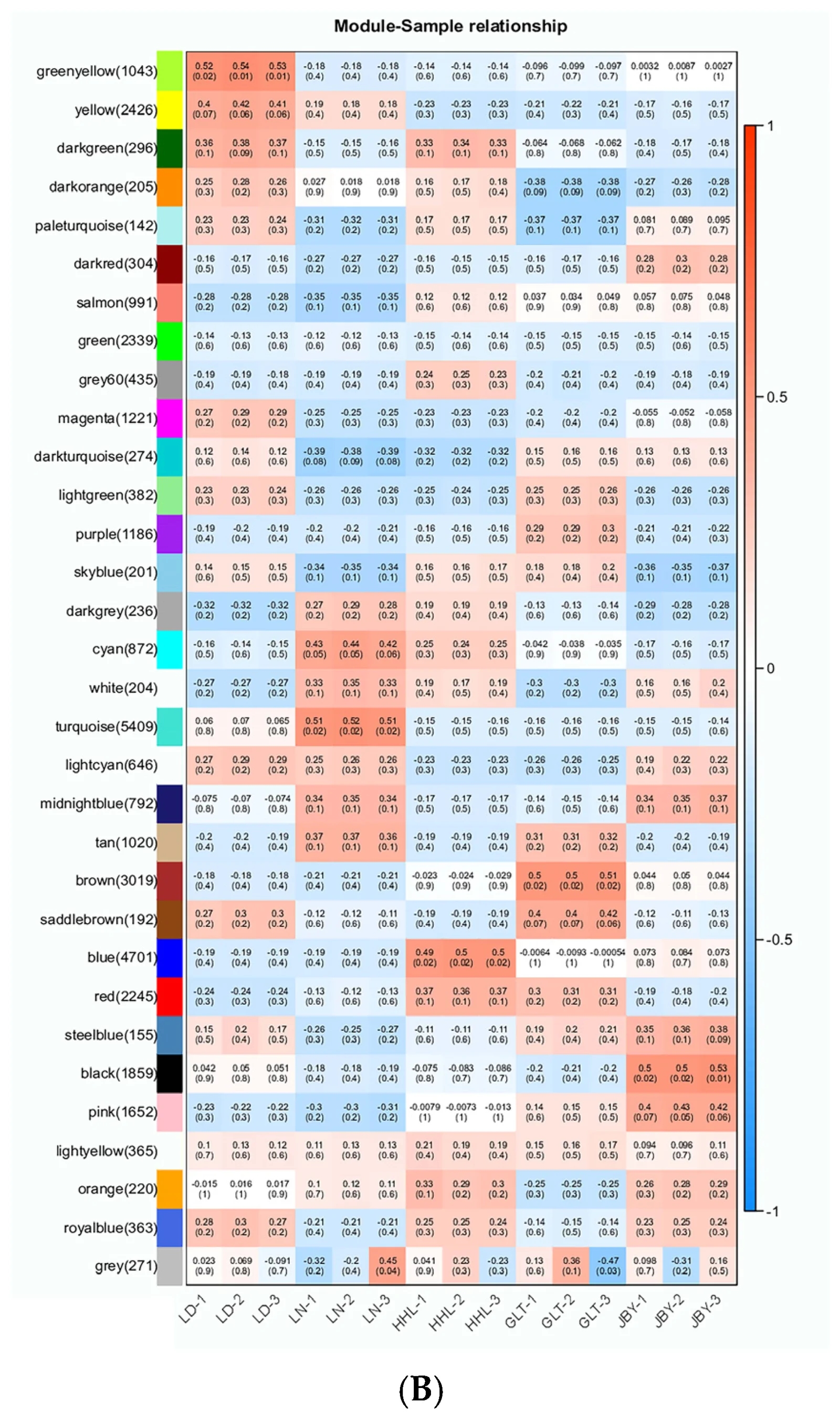
Weighted gene co-expression network analysis (WGCNA) of *K. blossfeldiana* with different flower colors. (**A**) Cluster dendrogram and module assignment of WGCNA. The module merging threshold is set to 0.25, and the minimum number of genes per module is 50. (**B**) Module–sample correlation heatmap showing the relationship between each module and flower color cultivars. The *x*-axis represents samples, while the *y*-axis represents modules. The number of module genes is indicated in parentheses. In the figure, the color intensity indicates the strength of the correlation, with red denoting a positive correlation and blue denoting a negative correlation.

Weighted gene co-expression network analysis (WGCNA) was conducted on the samples to identify the key gene modules associated with flower color formation. A scale-free network was established under a soft threshold β = 9, leading to the identification of 30 gene co-expression modules, each of which contained between 155 and 5409 genes (Figure 10A). Module–trait correlation analysis indicated significant associations between different modules and specific flower color cultivars (Figure 10B). Among these modules, the green–yellow module exhibited a strong positive correlation with LD, the blue module with HHL, the turquoise module with LN, the black module with JBY, and the brown module with GLT. These modules are regarded as potential regulators of flower color.

### 3.7. qPCR Validation of Core Pigment Pathway Genes

**Figure 11:**
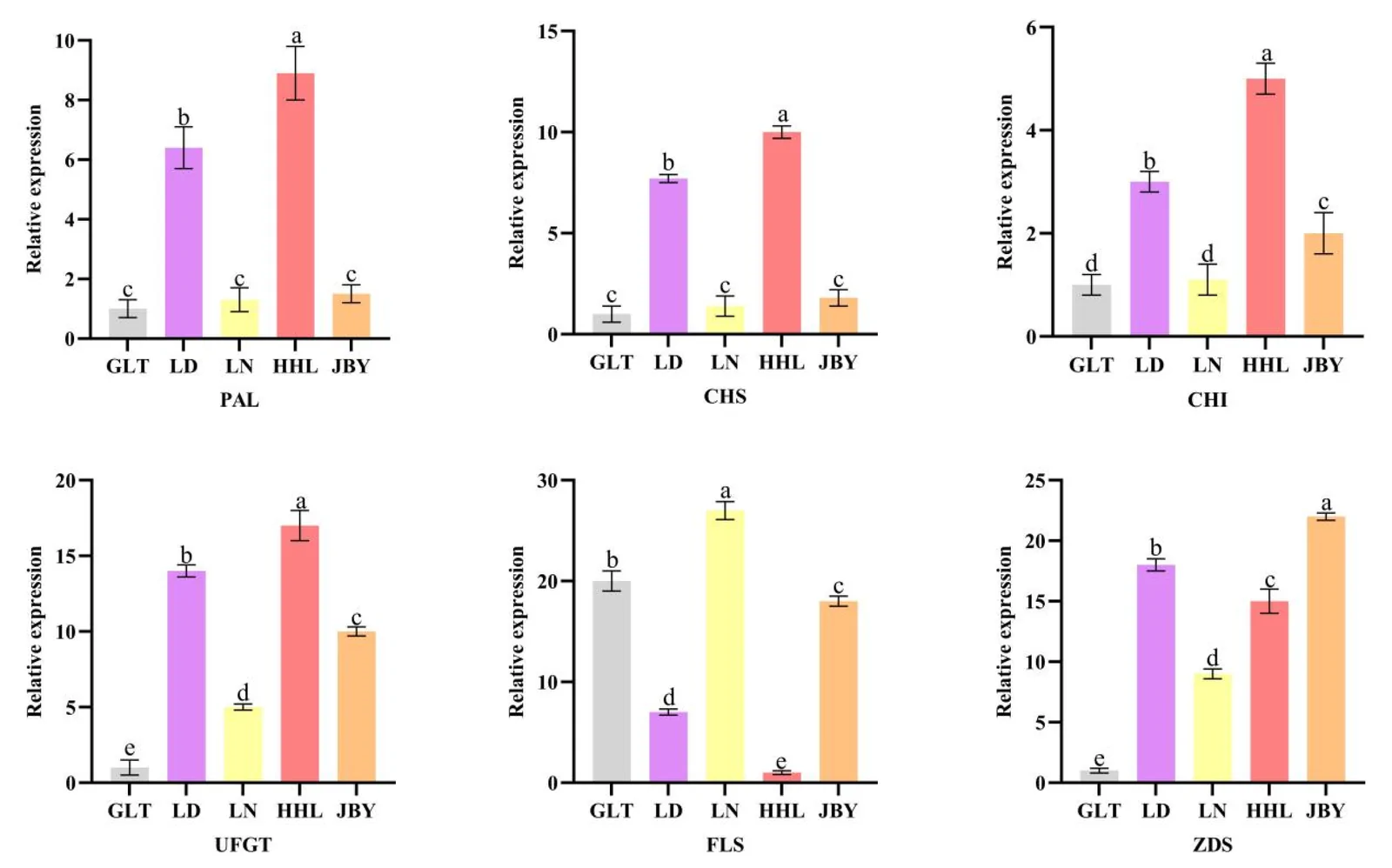
Relative expression of key genes associated with flower color in *K. blossfeldiana*. Three biological replicates were set up. One-way analysis of variance (ANOVA) and Duncan’s method were used to analyze the significance of data differences. The different letters within a column marked significant differences (*p* < 0.05).

To validate the reliability of the RNA-seq data, this study carefully selected six key genes associated with pigment biosynthesis for real-time quantitative PCR (qRT-PCR) verification: PAL, CHS, CHI, FLS, UFGT, and ZDS. These genes respectively represent crucial nodes in the phenylpropanoid–flavonoid pathway, flavonol pathway, and carotenoid pathway. The relative expression levels of these six genes measured by qRT-PCR showed that PAL, CHS, CHI, and UFGT exhibited notably higher expression levels in LD and HHL compared to GLT, which was consistent with the substantial accumulation of anthocyanins in these two red-colored cultivars. FLS showed relatively higher expression in GLT and LN, indicating that the metabolic flux might be directed toward flavonol synthesis in non-red or light-colored cultivars. ZDS displayed high expression in HHL, LD, and JBY, which is in alignment with the carotenoid measurement data (Figure 11). These qPCR results further corroborate the transcriptome findings and lend support to the proposed model of pathway competition and metabolic flux distribution.

## 4. Discussion

Feng et al. initially elucidated the molecular mechanism underlying the red–yellow flower color variation within a single bicolor *K. blossfeldiana* cultivar [20]. Nevertheless, this study was confined to the spatial heterogeneity within the cultivar and consequently could not reveal the regulatory logic underlying systematic divergence among cultivars [20]. Multi-omics integration analysis has emerged as the mainstream method for deciphering the mechanisms of flower color formation in ornamental plants [34,40]. This study developed an integrated metabolomics and transcriptomics analytical framework across five independent cultivars (white GLT, yellow LN, orange–yellow JBY, and red/pink LD and HHL), systematically depicting the differentiation patterns of flower color diversity in *K. blossfeldiana*.

Integrated metabolomics and transcriptomics KEGG analysis indicated that, when compared with GLT serving as the control, all four groups exhibited significant enrichment in phenylpropanoid metabolism and the flavonoid and flavonol biosynthesis pathways. Furthermore, 22 differentially accumulated flavonoid metabolites were commonly found across the four groups, encompassing flavonols, flavones, phenolic acid derivatives, and coumarins. This finding suggests that floral color diversity is established on a shared basis of flavonoid backbone synthesis. Downstream branching demonstrated distinct divergence among cultivars: UFGT was significantly up-regulated in LD and HHL, which promoted anthocyanin glycosylation, while FLS was dominant in GLT and LN, directing metabolic flux toward flavonol synthesis. This “shared upstream, divergent downstream” pattern is in line with the reports on color variation in water lilies [13], rhododendrons [37], and chrysanthemums [39].

The metabolism of carotenoids demonstrated an unanticipated pattern of differentiation. The LD and HHL cultivars shared 40 differentially expressed carotenoid metabolites, which implies that purple/red cultivars share a similar metabolic allocation strategy during carotenoid remodeling. In contrast, the yellow cultivar LN exhibited only 14 differentially expressed carotenoid metabolites, sharing 13 with the orange–yellow cultivar JBY. This number is significantly lower than the 41 of JBY and the 45 of HHL, indicating that LN has a highly restricted carotenoid differential profile. This finding contradicts the classical assumption that “yellow petals are dominated by carotenoids” [41,42]. It is likely that the yellow color in LN depends more on the flux distribution via the flavonol pathway rather than on substantial activation of the carotenoid pathway. Moreover, the orange–yellow differential profile of JBY extensively overlapped with those of LD and HHL, and no independent orange metabolic signature was detected. This suggests that its color may stem from the synergistic contributions of anthocyanins and carotenoids rather than being determined by a single pathway alone [43,44].

WGCNA identified 30 co-expression modules, and each cultivar was associated with distinct modules. This finding suggests that the diversification of flower color is driven by cultivar-specific transcriptional networks rather than the continuous variation in single genes. This precise correspondence between modules and traits is consistent with multi-omics studies conducted on ornamental plants such as chrysanthemums [45]. However, current predictions are still limited to the correlation level, and the causal regulatory roles of candidate modules need to be validated through transgenic experiments. qPCR further verified the expression patterns of six genes, namely PAL, CHS, CHI, FLS, UFGT, and ZDS, offering independent experimental evidence for the reliability of RNA-seq data and supporting the transcriptomic and metabolomic findings.

In summary, the color diversity of *K. blossfeldiana* is determined by both the general activation of flavonoid backbone synthesis and the cultivar-specific differentiation of downstream branches, whereas carotenoids participate in regulation via the differential accumulation of xanthophyll esters. Although this study has preliminarily revealed the differentiation patterns through the integration of metabolomics, transcriptomics, and network analysis, the functional validation of candidate modules and the dynamic metabolic flux tracking still necessitate further investigation through transgenic approaches and stage-specific sampling.

## 5. Conclusions

This study establishes a multi-omics comparative framework to dissect the metabolic basis of petal color variation in *K. blossfeldiana*. The findings indicate that flower color diversity arises from coordinated flux partitioning between flavonoid and carotenoid networks. A metabolic relay pattern is detected in the phenylpropanoid–flavonoid axis: shared upstream activation feeds divergent downstream branches, with UFGT-directed anthocyanin glycosylation characterizing red and purple–red phenotypes, and FLS-driven flavonol accumulation predominating in white and yellow cultivars. The yellow cultivar exhibits a restricted carotenoid profile and relies on flavonol flux for coloration, suggesting that yellow petal pigmentation is not exclusively carotenoid-dependent. Orange coloration appears to stem from the synergistic accumulation of anthocyanins and carotenoids. Weighted gene co-expression network analysis identifies transcriptional modules tightly associated with specific color phenotypes, and qPCR validates the expression divergence of six key structural genes (PAL, CHS, CHI, FLS, UFGT, and ZDS). These results support a pathway crosstalk model for flower color formation and provide candidate targets for molecular breeding in ornamental plants.

## Figures and Tables

**Table 1:** Types and quantities of metabolites detected in five cultivars of *K. blossfeldiana*.

Type	Subcategory	Quantity
Carotenoids	Xanthophylls	64
	Carotenes	8
	Total	72
Flavonoids	Flavonols	44
	Flavones	29
	Flavanones	11
	Flavanols	9
	Flavanonols	8
	Coumarins	8
	Phenylpropionic acids	6
	Chalcones	4
	Flavone glycosides	4
	Isoflavanones	3
	Benzoic acid derivatives	10
	Phenonic acids	2
	Stilbenes	2
	Aromatic aldehydes	2
	Anthocyanins	1
	Xanthones	1
	Tannins	1
	Total	168

## Data Availability

The datasets generated and/or analyzed during the current study are available in the NCBI (National Center for Biotechnology Information: https://www.ncbi.nlm.nih.gov, accessed on 6 July 2026) repository, PRJNA1490288.

## Supplementary Materials

The following supporting information can be downloaded at: https://www.mdpi.com/article/10.3390/genes17080934/s1, Figure S1: TIC chromatogram of QC samples for flavonoids and carotenoids; Figure S2: Transcriptome sequencing, assembly, and sample clustering in five *K. blossfeldiana* cultivars; Table S1: Summary of RNA-seq data quality for petal transcriptomes of five *K. blossfeldiana* cultivars; Table S2: Primers used in the qRT-PCR.

## Abbreviations

The following abbreviations are used in this manuscript:

PAL	phenylalanine ammonia-lyase
C4H	cinnamate 4-hydroxylase
CHS	chalcone synthase
CHI	chalcone isomerase
F3H	flavanone 3-hydroxylase
DFR	dihydroflavonol 4-reductase
ANS	anthocyanidin synthase
UFGT	UDP-glucose flavonoid 3-O-glucosyltransferase
FLS	flavonol synthase
IPP	isopentenyl diphosphate
DMAPP	dimethylallyl diphosphate
PSY	phytoene synthase
PDS	phytoene desaturase
ZDS	ζ-carotene desaturase
LCY	lycopene cyclase
MBW	MYB-bHLH-WD40
WD40	WD40 repeat domain
UPLC-MS	ultra-performance liquid chromatography-mass spectrometry
UPLC-HESI-MS	ultra-performance liquid chromatography with heated electrospray ionization mass spectrometry
UPLC-MS/MS	ultra-performance liquid chromatography-tandem mass spectrometry
ESI	electrospray ionization
APCI	atmospheric pressure chemical ionization
MRM	multiple reaction monitoring
CID	collision-induced dissociation
TEM	turbo electrospray mode
MS	mass spectrometry
LC	liquid chromatography
HPLC	high-performance liquid chromatography
UV	ultraviolet
MTBE	methyl tert-butyl ether
CUR	curtain gas
GS1	gas source 1
GS2	gas source 2
MWDB	MetWare self-built database
PCA	principal component analysis
ANOVA	analysis of variance
QC	quality control
SD	standard deviation
WGCNA	weighted gene co-expression network analysis
GO	Gene Ontology
BP	biological process
MF	molecular function
CC	cellular component
KEGG	Kyoto Encyclopedia of Genes and Genomes
RNA-seq	RNA sequencing
qRT-PCR	quantitative reverse transcription PCR
PCR	polymerase chain reaction
FPKM	fragments per kilobase of transcript per million mapped reads
RIN	RNA integrity number
