# Supplementary material for: Pathway Crosstalk and Metabolic Relay in the Phenylpropanoid–Carotenoid Axis Orchestrate Petal Coloration in Kalanchoe blossfeldiana
Source: Genes (Basel). 2026 Aug 11;17(8):934. doi: 10.3390/genes17080934 (PMC13511887; doi:10.3390/genes17080934)
Supplement: Supplementary file 1 [file genes-17-00934-s001.zip › Supplementary materials DOC version (including figure legend).pdf]

Supplementary materials

A

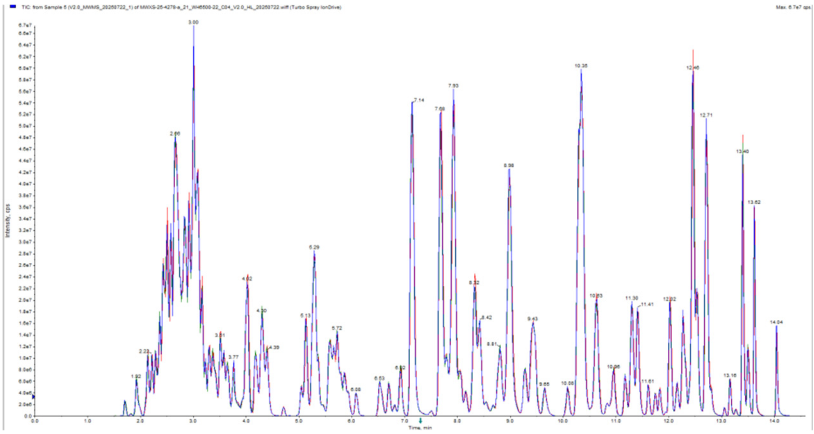

B

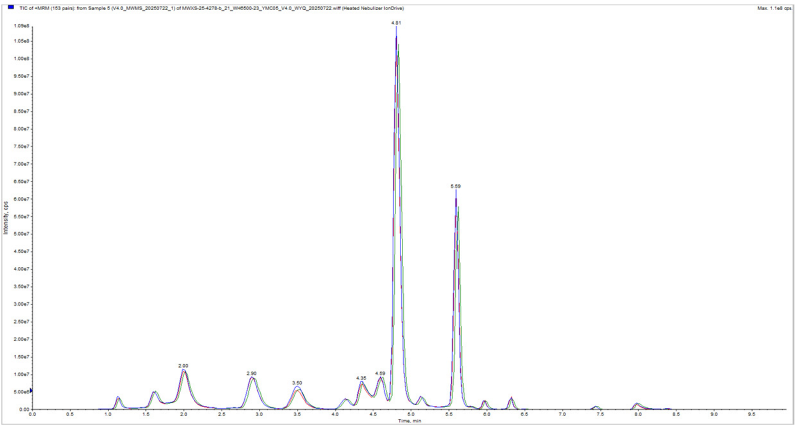

Figure S1 TIC chromatogram of QC samples for flavonoids and carotenoids. **(A)** TIC chromatogram of QC sample for flavonoids. **(B)** TIC chromatogram of carotenoid QC sample.The high overlap of total ion current curves in metabolite detection, which indicates consistent retention times and peak intensities, suggests good signal stability of the mass spectrometer when analyzing the same sample at different times.

A

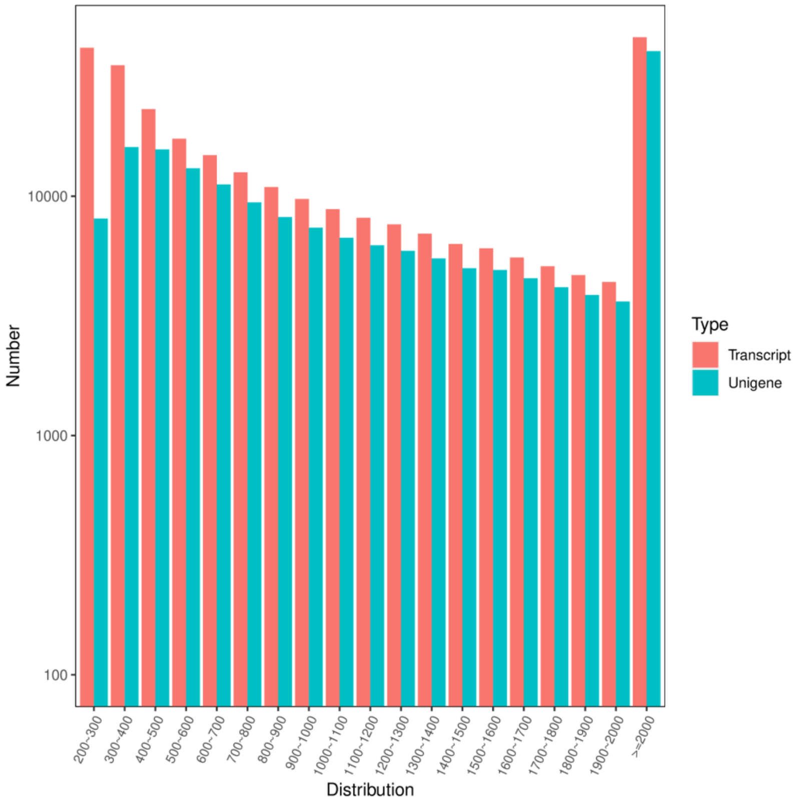

Number
10000
1000
100
200~300
300~400
400~500
500~600
600~700
700~800
800~900
900~1000
1000~1100
1100~1200
1200~1300
1300~1400
1400~1500
1500~1600
1600~1700
1700~1800
1800~1900
1900~2000
>=2000
Distribution
Type
Transcript
Unigene

B

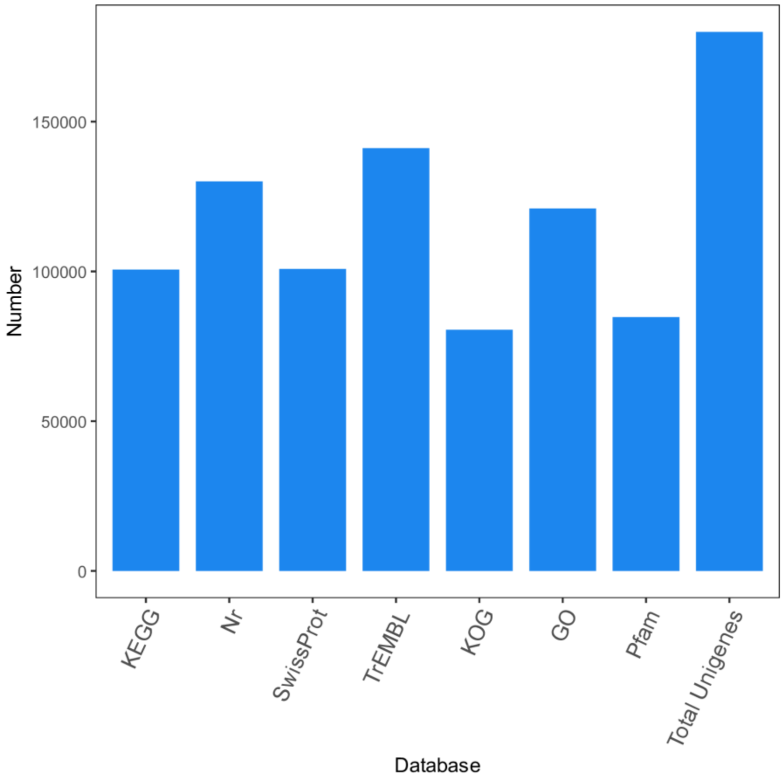

Number
150000
100000
50000
0
KEGG
Nr
SwissProt
TrEMBL
KOG
GO
Pfam
Total Unigenes
Database

C

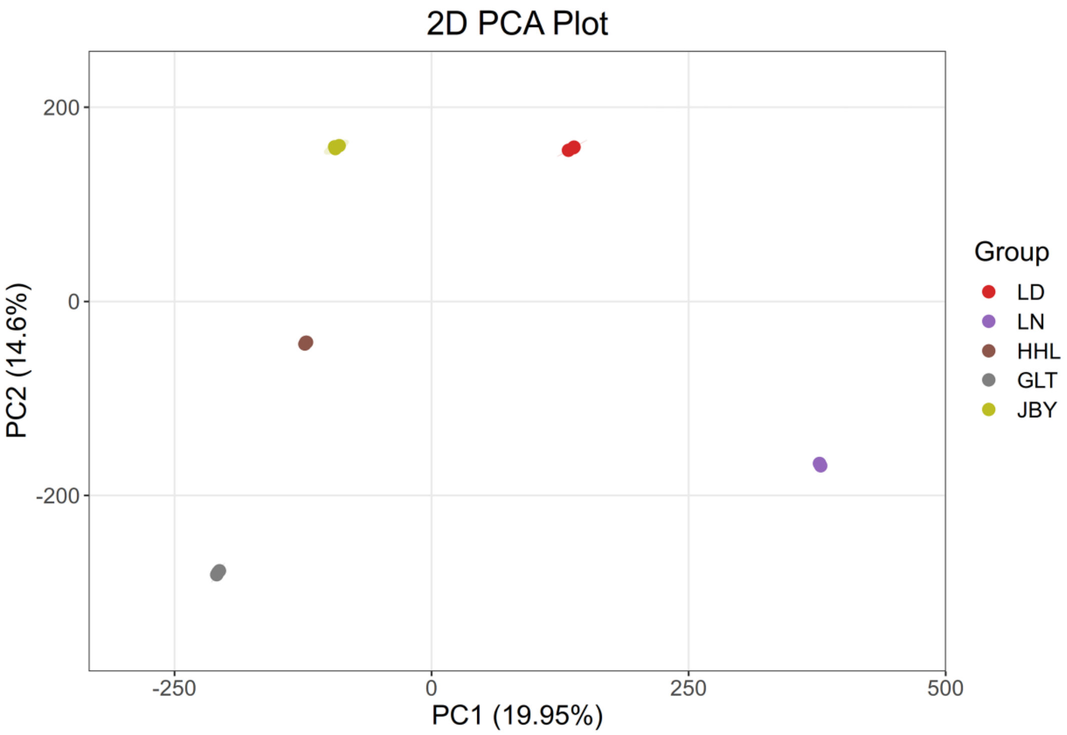

Figure S2 Transcriptome sequencing, assembly, and sample clustering in five *K. blossfeldiana* cultivars. **(A)** Length distribution of assembled transcripts and unigenes. **(B)** Bar chart of functional annotation across seven public databases. **(C)** PCA of all 15 samples based on FPKM values.

Table S1 Summary of RNA-seq data quality for petal transcriptomes of five *Kalanchoe blossfeldiana* cultivars.

Sample	Group	Raw Reads	Raw Base(G)	Clean Reads	Clean Base(G)	Error Rate(%)	Q20(%)	Q30(%)	GC Content(%)
GLT-1	GLT	60988312	9.15	59681484	8.95	0.02	99.17	97.1	47.95
GLT-2	GLT	49331606	7.4	48297376	7.24	0.02	99.06	96.66	48
GLT-3	GLT	55304064	8.3	54434716	8.17	0.02	98.96	96.32	47.92
HHL-1	HHL	50660222	7.6	50040316	7.51	0.02	99.12	96.87	48.06
HHL-2	HHL	61441914	9.22	60621368	9.09	0.02	99.13	96.95	48.3
HHL-3	HHL	50489436	7.57	49822610	7.47	0.02	99.02	96.51	48.34
JBY-1	JBY	55822244	8.37	54926224	8.24	0.02	99.1	96.78	47.99
JBY-2	JBY	56915440	8.54	56339040	8.45	0.02	99.02	96.51	47.98
JBY-3	JBY	47923666	7.19	47145962	7.07	0.02	99.08	96.74	47.83
LD-1	LD	50551904	7.58	49722140	7.46	0.02	99.07	96.69	48.3
LD-2	LD	50299474	7.54	49200418	7.38	0.02	98.98	96.4	48.09
LD-3	LD	49558268	7.43	48891740	7.33	0.02	99.02	96.53	48.08
LN-1	LN	56476988	8.47	55421282	8.31	0.02	99.01	96.48	48.02
LN-2	LN	54460610	8.17	53575650	8.04	0.02	99.01	96.48	48.01
LN-3	LN	55368856	8.31	54332058	8.15	0.02	99.01	96.51	47.98

Table S2 Primers used in the qRT-PCR

Name	Primer sequence
KbActin	F: CGGAATCGCTGACAGAATGA<br>R: GAAGGTGCTGAGAGATGCCA
KbPAL	F: AGAATACACCAAGTCCGCTGAG<br>R: CAGTCTCCTTCTCTTAGTTTGCCT
KbCHS	F: GACACCCATTTGGACAGCCT<br>R: CGAGATGCCGAGTGGTTTGA
KbCHI	F: AGCAATACTCAGAAAAGGTGGC<br>R: CCCTCTTTTGGTATCTCATCGTGT
KbFLS	F: TGGAGGAGAAGATTGAGTGGATAG<br>R: GACCCAACCCTTGTGATAGTAGC
KbUFGT	F: ATTGTGAGCGGTGAGCAGGT<br>R: CCTGAGACCACTGATGAACTGCT
KbZDS	F: GGGTTCCCCAGATGTTTATTTG<br>R: CTCTCCATTGCGAAGGAAGTAA
